# YAP and TAZ maintain PROX1 expression in the developing lymphatic and lymphovenous valves in response to VEGF-C signaling

**DOI:** 10.1242/dev.195453

**Published:** 2020-12-13

**Authors:** Boksik Cha, Yen-Chun Ho, Xin Geng, Md. Riaj Mahamud, Lijuan Chen, Yeunhee Kim, Dongwon Choi, Tae Hoon Kim, Gwendalyn J. Randolph, Xinwei Cao, Hong Chen, R. Sathish Srinivasan

**Affiliations:** 1 Cardiovascular Biology Research Program, Oklahoma Medical Research Foundation, Oklahoma City, OK 73104, USA; 2 Daegu Gyeongbuk Medical Innovation Foundation, Daegu 41061, Republic of Korea; 3 Department of Biological Sciences and Center for Systems Biology, The University of Texas at Dallas, Richardson, TX 75080, USA; 4 Keck School of Medicine, University of Southern California, Los Angeles, CA 90033, USA; 5 Department of Pathology and Immunology, Washington University School of Medicine, St Louis, MO 63110, USA; 6 Department of Developmental Neurobiology, St. Jude Children's Research Hospital, Memphis, TN 38105, USA; 7 Vascular Biology Program, Boston Children's Hospital, Boston, MA 02115, USA; 8 Department of Cell Biology, University of Oklahoma Health Sciences Center, Oklahoma City, OK 73117, USA

**Keywords:** PROX1, Lymphovenous valves, Valves, VEGF-C, YAP, TAZ

## Abstract

Lymphatic vasculature is an integral part of digestive, immune and circulatory systems. The homeobox transcription factor PROX1 is necessary for the development of lymphatic vessels, lymphatic valves (LVs) and lymphovenous valves (LVVs). We and others previously reported a feedback loop between PROX1 and vascular endothelial growth factor-C (VEGF-C) signaling. PROX1 promotes the expression of the VEGF-C receptor VEGFR3 in lymphatic endothelial cells (LECs). In turn, VEGF-C signaling maintains PROX1 expression in LECs. However, the mechanisms of PROX1/VEGF-C feedback loop remain poorly understood. Whether VEGF-C signaling is necessary for LV and LVV development is also unknown. Here, we report for the first time that VEGF-C signaling is necessary for valve morphogenesis. We have also discovered that the transcriptional co-activators YAP and TAZ are required to maintain PROX1 expression in LVs and LVVs in response to VEGF-C signaling. Deletion of *Yap* and *Taz* in the lymphatic vasculature of mouse embryos did not affect the formation of LVs or LVVs, but resulted in the degeneration of these structures. Our results have identified VEGF-C, YAP and TAZ as a crucial molecular pathway in valve development.

## Abstract (precis)

**Summary:** YAP and TAZ mediate the positive feedback loop between PROX1 and VEGF-C signaling in the developing lymphatic and lymphovenous valves.

## INTRODUCTION

Lymphatic vasculature is an integral part of digestive, immune and circulatory systems of vertebrates. Lymphatic vessels absorb interstitial fluid and return it to blood circulation (Adams and Alitalo, 2007). In addition, specialized lymphatic vessels of the gut known as lacteals absorb digested lipids. Lymphatic vessels regulate immune response by transporting immune cells from tissues to lymph nodes. Lymphatic vessels also resolve inflammation by clearing extravasated fluids and immune cells at the site of inflammation. Defects in lymphatic vasculature can cause lymphedema, a disease in which tissues swell due to excessive fluid accumulation. Currently, we lack approaches to treat lymphedema, which has common comorbidities such as infections, inflammation, obesity and fibrosis (Tammela and Alitalo, 2010). Improved understanding of the mechanisms that regulate lymphatic vascular development could provide innovative opportunities for treating lymphedema and other lymphatic vascular disorders.

Lymphatic vasculature is arranged in a hierarchical manner. Blind-ended lymphatic capillaries collect interstitial fluid, immune cells or digested lipids (simply called as lymph), and transport them to collecting lymphatic vessels. Lymphatic valves (LVs) within the collecting lymphatic vessels regulate the unidirectional flow of lymph and prevent backflow. Finally, lymph travels through the thoracic duct or the right lymphatic duct and returns to blood circulation via two pairs of lymphovenous valves (LVVs) located bilaterally at the intersection of jugular and subclavian veins (Geng et al., 2016; Srinivasan and Oliver, 2011).

Lymphatic vessels, LVs and LVVs are established from lymphatic endothelial cells (LECs) that originate predominantly from the embryonic veins (Pichol-Thievend et al., 2018; Srinivasan et al., 2007). Other sources could make minor contribution to the lymphatic vasculature in a tissue-specific manner (Gancz et al., 2019; Lioux et al., 2020; Martinez-Corral et al., 2015; Maruyama et al., 2019; Stanczuk et al., 2015). LEC progenitors are specified in the embryonic veins by the transcription factor PROX1, which activates the expression of molecules such as the receptor tyrosine kinase VEGFR3 and the glycoprotein podoplanin (Hong et al., 2002; Petrova et al., 2002; Srinivasan et al., 2014; Wigle et al., 2002; Wigle and Oliver, 1999). The potent lymphangiogenic molecule VEGFC associates with VEGFR3 to promote LEC migration from the veins to form the lymph sacs (Karkkainen et al., 2004). A subset of LEC progenitors do not upregulate VEGFR3 expression and remain in the veins to form two pairs of LVVs through which lymph sacs interact with the veins (Geng et al., 2016; Srinivasan and Oliver, 2011).

Lymphatic vessels sprout from the lymph sacs to form the primitive lymphatic plexus in various tissues. Subsequent maturation of the lymphatic plexus results in the formation of lymphatic capillaries and collecting lymphatic vessels (Norrmén et al., 2009). LVs develop within the collecting lymphatic vessels (Bazigou et al., 2009; Norrmén et al., 2009; Petrova et al., 2004). Maturation of lymphatic plexus and the formation of valves are regulated by various signaling molecules such as VEGF-C, neuropilin 2, Wnt/β-catenin, S1PR1, BMP9, ephrin B2, EPH-B4, plexins and semaphorins (Bouvrée et al., 2012; Cha et al., 2016, 2018; Geng et al., 2020; Jurisic et al., 2012; Levet et al., 2013; Liu et al., 2014, 2016; Makinen et al., 2005; Martin-Almedina et al., 2016; Nurmi et al., 2015; Yuan et al., 2002). Shear stress generated by lymph flow also plays a deterministic role in lymphatic vascular morphogenesis (Cha et al., 2016, 2018; Choi et al., 2019, 2017a,b; Geng et al., 2020; Liu et al., 2014; Sabine et al., 2012; Sweet et al., 2015; Wang et al., 2016). Despite these advances, our basic understanding of the mechanisms of lymphatic vascular development remain incomplete. How do the various signaling pathways coordinate with each other to regulate lymphatic vascular development in a precise spatiotemporal manner is not clear.

Hippo signaling controls cell proliferation, survival and differentiation during tissue development, regeneration and homeostasis (Zheng and Pan, 2019). The transcription co-factors YAP (Yes-associated protein) and TAZ (transcriptional co-activator with PDZ-binding motif) are key downstream effectors of the Hippo pathway. When Hippo signaling is ‘off’, non-phosphorylated YAP and TAZ accumulate in the nucleus and interact with transcription factors such as TEAD4, TBX5 and SMAD to induce target genes. Importantly, YAP and TAZ function as gatekeepers of biochemical and mechanical signaling pathways such as Wnt/β-catenin, TGFβ, GPCR, ECM stiffness and shear stress (Piccolo et al., 2014). In the blood vasculature, YAP and TAZ promote retinal angiogenesis and maturation of endothelial cell barrier by regulating VEGF, BMP, Rho GTPase and biomechanical signals (Kim et al., 2017; Neto et al., 2018; Sakabe et al., 2017; Wang et al., 2017). Intriguingly, YAP and TAZ inhibit angiogenesis within bones by negatively regulating HIF pathway (Sivaraj et al., 2020). The co-repressor activity of the YAP/TAZ/TEAD4/NuRD complex is likely responsible for the transcriptional inhibitory function of YAP and TAZ (Kim et al., 2015).

We are starting to understand the roles of YAP and TAZ in the mammalian lymphatic vasculature. TAZ and the ECM protein CTGF, which is a canonical target of YAP and TAZ, are expressed in LVs, but not in non-valvular LECs (Sabine et al., 2015). Conditional deletion of *Yap* and *Taz* from mouse LECs disrupts the maturation of the lymphatic vessels and results in the absence of LVs (Cho et al., 2019). Maturation of collecting lymphatic vessels involves the pruning of excessive branches and downregulation of PROX1 and VEGFR3 expression (Norrmén et al., 2009; Petrova et al., 2004). PROX1 fails to undergo downregulation in the absence of YAP and TAZ. Furthermore, VEGF-C inhibits YAP and TAZ activity to promote PROX1 expression in primary human LECs (Cho et al., 2019). The downstream side of LVs experience oscillatory shear stress (OSS), and OSS enhances the expression of the lymphedema-associated transcription factor FOXC2 that is necessary for LV development (Petrova et al., 2004; Sabine et al., 2012). Importantly, FOXC2 inhibits OSS-induced YAP and TAZ activity in primary human LECs (HLECs) (Sabine et al., 2015). Furthermore, stiff ECM could also enhance YAP and TAZ activity in HLECs (Frye et al., 2018). Despite these findings, whether YAP and TAZ are required for the formation of LVs and LVVs or for their maintenance is not known. To address this important issue, we investigated the mechanisms of YAP and TAZ activity during LV and LVV development in mice.

## RESULTS

### YAP and TAZ are activated during valve maturation

LVV development starts at embryonic day (E) 12.0 in mice (Geng et al., 2016; Srinivasan and Oliver, 2011). PROX1, FOXC2 and GATA2 are upregulated in LVV-ECs, which delaminate from the luminal side of the jugular vein. LVV-ECs rapidly reaggregate and elongate to form mature LVVs at E12.5; after this stage, the changes within the valves are subtle.

We performed immunohistochemistry to investigate the nuclear localization of YAP and TAZ, and the expression of their target molecule CTGF in LVVs starting from E12.0. YAP, TAZ and CTGF displayed low expression in LVV-ECs until E15.5 (Fig. S1A,B). At E15.5, YAP and TAZ were detected in both the cytoplasm and nucleus of LVV-ECs (Fig. S1C). CTGF was observed in the ECM of LVV-ECs (Fig. S1D). By E16.5, YAP and TAZ were predominantly nuclear in LVV-ECs (Fig. S1E), and CTGF remains enriched in LVVs (Fig. S1F). Semi-quantitative measurement of YAP, TAZ and CTGF expression indicates that their expression in LVVs increases significantly between E13.5 and E16.5 (Fig. S1G,H).

**Fig. 1.**
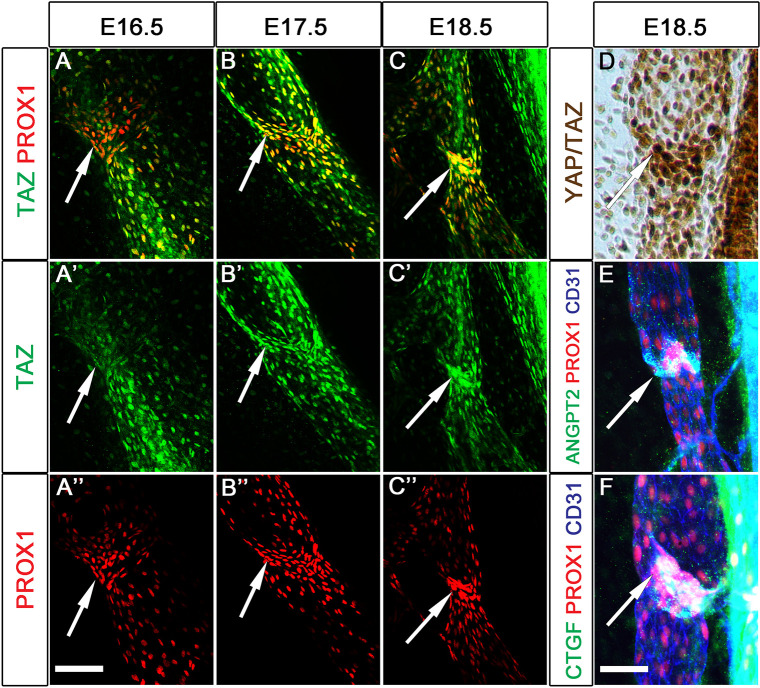
**YAP and TAZ activity is upregulated in LVs undergoing maturation.** (A-C″) Mesenteric lymphatic vessels from E16.5 (A-A″), E17.5 (B-B″) and E18.5 (C-C″) embryos were analyzed for the expression of TAZ. A gradual increase in the expression of TAZ was observed in the LVs from E16.5 to E18.5 (arrows). (D-F) Immunohistochemical analysis of E18.5 mesenteric vessels revealed strong expression of YAP and TAZ (D), and their targets ANGPT2 (E) and CTGF (F) in the LVs (arrows). *n*=3 embryos per stage per antibody. Scale bars: 200 µm in A-C″; 100 µm in D-F.

YAP and TAZ were similarly expressed in mature mesenteric LVs (Fig. 1). Although YAP and TAZ were not expressed in newly formed LV-ECs at E16.5 (Fig. 1A), they were detectable in LV-ECs at E17.5 (Fig. 1B) and were strongly expressed by E18.5 (Fig. 1C,D). Angiopoietin 2 (ANGPT2), another target of YAP and TAZ (Choi et al., 2015; He et al., 2018; Kim et al., 2017), was also observed in LV-ECs at E18.5 (Fig. 1E). In addition, CTGF was expressed in mature LVs as reported previously (Fig. 1F) (Sabine et al., 2015). Together, these results suggest that the activity of YAP and TAZ gradually increases in developing LVs and LVVs.

### YAP and TAZ are required to maintain valvular endothelial cells

**Fig. 2.**
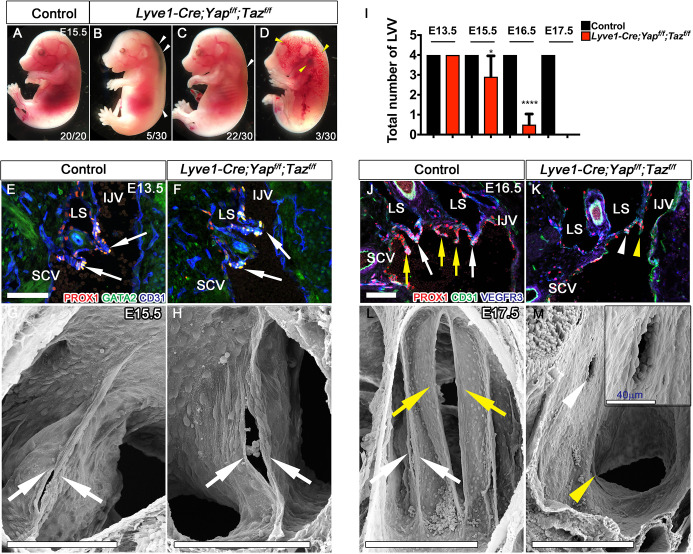
**YAP and TAZ are required for the maintenance of LVVs.** (A) Control E15.5 mouse embryo. (B-D) A subset of E15.5 *Lyve1-Cre;Yap^f/f^;Taz^f/f^* embryos developed edema (B, arrowheads) or blood-filled lymphatic vessels (D, arrowheads). The rest of the embryos had mild edema (C, arrowhead) or no obvious defects. The number of embryos analyzed in total and the number of embryos that had the represented phenotype are indicated in each panel. (E-H) Immunohistochemistry or scanning electron microscopy was used to analyze the LVVs of E13.5 (E,F) and E15.5 (G,H) embryos. LVVs (arrows) were observed in both control (E,G) and *Lyve1-Cre;Yap^f/f^;Taz^f/f^* (F,H) embryos. (I) Every embryo has four LVVs. We analyzed *n*=4 embryos/genotype/stage and calculated the average number of LVVs per embryo. (J-M) E16.5 (J,K) and E17.5 (L,M) embryos were analyzed by immunohistochemistry or scanning electron microscopy. LVVs (white arrows) and venous valves (yellow arrows) were seen in control embryos (J,L). In contrast, only few PROX1^+^ valvular endothelial cells were seen in E16.5 *Lyve1-Cre;Yap^f/f^;Taz^f/f^* embryos (K, arrowheads). Scanning electron microscopy revealed abnormal holes in place of LVVs (M, white arrowhead) and a few venous valve-forming endothelial cells (M, yellow arrowhead). *P<0.05, ****P<0.0001. Data are mean±s.e.m. Scale bars: 100 µm in E-H,J,K; 200 µm in L,M.

To determine the function of YAP and TAZ during lymphangiogenesis, we used *Lyve1-Cre* to conditionally and constitutively delete YAP/TAZ in the LEC progenitors from E10.5 in mouse embryos (Pham et al., 2010; Xin et al., 2013, 2011). *Lyve1-Cre;Yap^flox/flox^* and *Lyve1-Cre;Taz^flox/flox^* mice were born alive and were phenotypically normal. However, we did not obtain *Lyve1-Cre;Yap^flox/flox^;Taz^flox/flox^* (referred to as *Yap/Taz^LECKO^*) pups. We analyzed *Yap/Taz^LECKO^* mouse embryos at various stages. Although E14.5 *Yap/Taz^LECKO^* embryos did not show any gross phenotypes, YAP and TAZ expression was strikingly downregulated in their LECs and LVV-ECs (Fig. S2). E15.5 *Yap/Taz^LECKO^* embryos showed edema with variable penetrance, with most embryos showing no obvious phenotype (Fig. 2A-D).

There are two pairs of bilaterally located LVVs in mammalian embryos (Geng et al., 2016; Srinivasan and Oliver, 2011). We detected four LVVs in all E13.5 and most E15.5 *Yap/Taz^LECKO^* embryos (Fig. 2E-H). The number of LVVs was slightly but significantly reduced in E15.5 *Yap/Taz^LECKO^* embryos compared with littermate controls (Fig. 2I). Strikingly, by E16.5 and E17.5, *Yap/Taz^LECKO^* embryos almost completely lacked LVVs (Fig. 2I-M). Unlike control E17.5 embryos, E17.5 *Yap/Taz^LECKO^* embryos displayed only a few LVV-ECs or small holes where LVVs normally form (Fig. 2M, white arrowhead and higher magnification inset). Venous valves that develop close to LVVs are a part of the blood vasculature, although they share the same molecular profile as LVVs (Bazigou et al., 2011; Geng et al., 2016; Munger et al., 2016, 2013; Srinivasan and Oliver, 2011). Consistent with *Lyve1-Cre* activity in embryonic veins, E17.5 *Yap/Taz^LECKO^* embryos also lacked venous valves (Fig. 2K,M, yellow arrowheads).

**Fig. 3.**
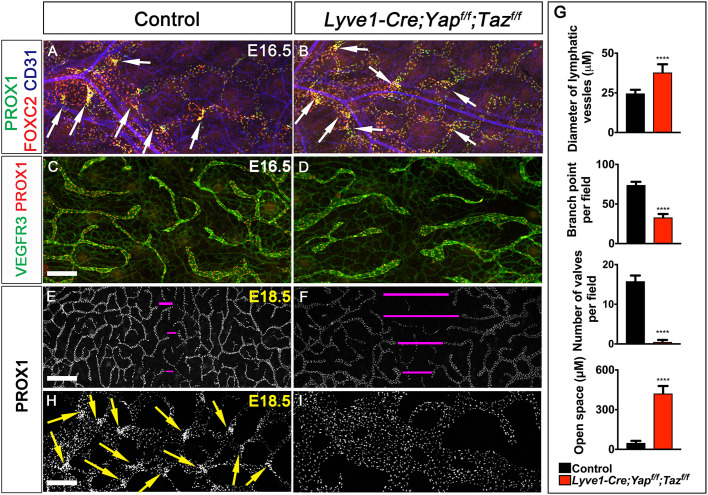
**YAP and TAZ are required for the maintenance of LVs.** The lymphatic vessels in the dorsal skin of E16.5 and E18.5 control and *Lyve1-Cre;Yap^f/f^;Taz^f/f^* embryos were analyzed by whole-mount immunohistochemistry. (A,B) LVs were observed in the collecting lymphatic vessels of E16.5 control and *Lyve1-Cre;Yap^f/f^;Taz^f/f^* embryos (arrows). (C,D) The migrating front of E16.5 control (C) and *Lyve1-Cre;Yap^f/f^;Taz^f/f^* (D) embryos appeared comparable. (E-G) At E18.5, the lymphatic vessels from the left and right sides have merged to form a network in control embryos (E). In contrast, huge gaps were observed in between the migrating fronts of E18.5 *Lyve1-Cre;Yap^f/f^;Taz^f/f^* embryos (F, magenta lines). The lymphatic vessels of mutant embryos were also dilated. The distance between the migrating fronts and the diameter of vessels are quantified in G. (H,I) LVs were observed in the collecting lymphatic vessels of E18.5 control embryos (H, yellow arrows). In contrast, the dilated lymphatic vessels of E18.5 *Lyve1-Cre;Yap^f/f^;Taz^f/f^* embryos lacked LVs (I). The various parameters of lymphatic vascular patterning were quantified and are plotted in G. *n*=4 embryos per each genotype. ****P<0.0001. Data are mean±s.e.m. Scale bars: 200 µm in A-D; 500 µm in E,F; 200 µm in H,I.

E16.5 *Yap/Taz^LECKO^* embryos lacked observable defects in the lymphatic vessels of the dorsal skin, and LV-forming endothelial cells were observed within their lymphatic vessels of the mutants (Fig. 3A,B, arrows). However, by E18.5, *Yap/Taz^LECKO^* embryos had defective lymphatic vessels that were more dilated, had fewer branch points and did not migrate sufficiently from the lateral edges (Fig. 3E-G). In addition, unlike control embryos (Fig. 3H, arrows), E18.5 *Yap/Taz^LECKO^* embryos no longer had any LVs (Fig. 3G,I).

The mesenteric lymphatic vessels of E18.5 *Yap/Taz^LECKO^* embryos were immature, as suggested by strong expression of LYVE1, VEGFR3 and PROX1 (Fig. S3). LVs were missing from these immature collecting vessels (Fig. S3C-G). However, the guts of *Yap/Taz^LECKO^* embryos were much smaller in size when compared with controls (Fig. S3E,F). This is likely due to the blood vascular defects caused by *Lyve1-Cre* expression in the blood vessels of the gut, as reported previously (Dellinger et al., 2013; Geng et al., 2020). Consequently, we are unable to conclude whether the defects in the mesenteric lymphatic vessels of *Yap/Taz^LECKO^* embryos are due to YAP and TAZ activity in LECs or to blood vascular endothelial cells. Nevertheless, *Lyve1-Cre* is specific to dermal lymphatic vasculature (Dellinger et al., 2013; Geng et al., 2020). Hence, based on our findings we conclude that YAP and TAZ are not required for the differentiation of valvular endothelial cells (LVVs and LVs). This conclusion coincides with the lack of YAP and TAZ expression and activity in the newly differentiated valvular endothelial cells. However, LVVs and LVs degenerate in the absence of YAP and TAZ.

### YAP and TAZ positively regulate PROX1 expression in primary human LECs

**Fig. 4.**
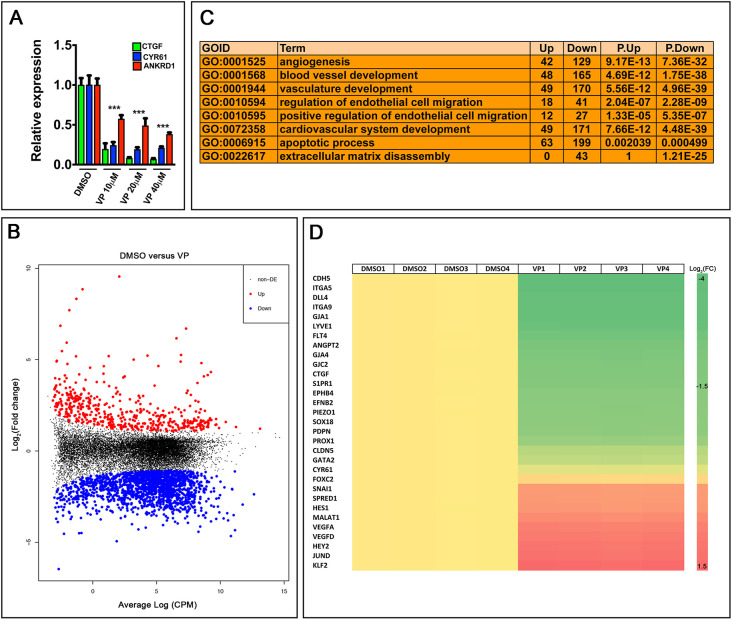
**YAP and TAZ regulate the expression of genes that are involved in lymphatic vascular development.** (A) Treatment of HLECs with VP resulted in the downregulation of YAP and TAZ target genes. (B) RNA-seq data were analyzed using a volcano plot to reveal the differentially expressed (DE) genes in VP-treated HLECs. (C) Gene ontology (GO) analysis revealed that several pathways that are crucial for vascular development were affected by VP treatment. (D) Heat map shows that the expression of several genes that are crucial for vascular development were affected by VP treatment. *n*=4 independent experiments per group.

The small molecule verteporfin (VP) disrupts the interaction between YAP, TAZ and TEAD, thereby inhibiting the transcriptional activity of YAP and TAZ. Primary human LECs (HLECs) treated with VP for 2 h showed reduced expression of the canonical YAP and TAZ target genes *CTGF* (*CCN2*), *CYR61* (*CCN1*) and *ANKRD1*, as expected (Fig. 4A). To comprehensively identify the transcriptional targets of the TEAD/YAP/TAZ complex, we treated HLECs with 20 µM VP for 2 h, extracted RNA and performed RNA-seq analysis. Based on the Log_2_(fold change) >1 that we set for differentially expressed genes, we determined that 794 genes were upregulated and 2161 genes were downregulated by VP [Fig. 4B and see data in the Dryad Digital Repository (Cha et al., 2020) and Table S1]. Gene ontology (GO) analysis showed that VP altered the expression of genes that regulate vascular development (Fig. 4C). Genes that regulate lymphangiogenesis, such as *PROX1*, *FLT4*, *ANGPT2* and *DLL4* were also dramatically downregulated in VP-treated HLECs (Fig. 4D).

**Fig. 5.**
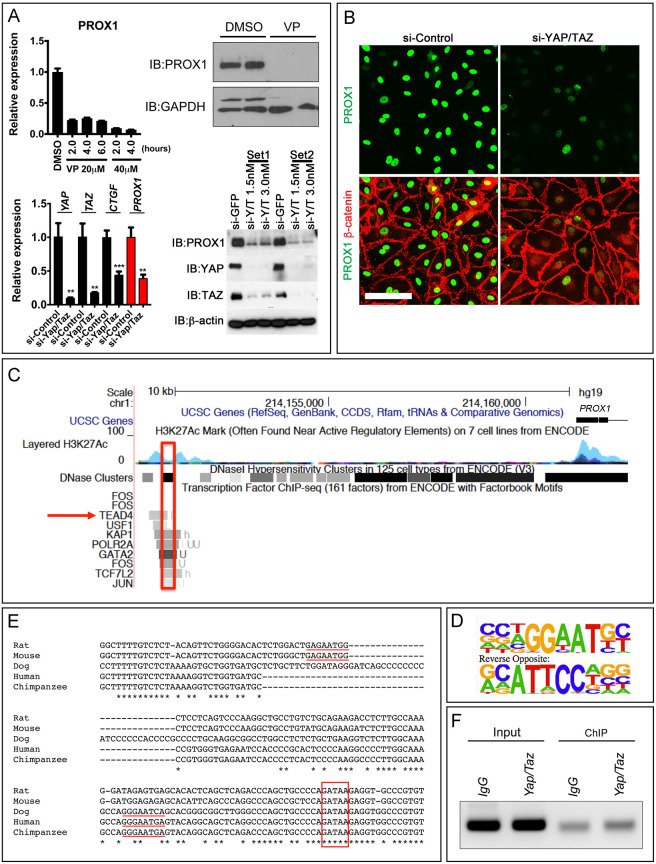
**YAP and TAZ regulate the expression of PROX1 in HLECs potentially through a conserved TEAD4-binding site.** (A) Inhibition of YAP and TAZ activity in HLECs by VP or siRNAs results in the downregulation of PROX1 at RNA and protein levels. IB, immunoblot. (B) Immunocytochemistry revealed that the expression of PROX1 is downregulated in HLECs by siRNAs targeting YAP and TAZ. (C) The PROX1 regulatory element was analyzed using the ENCODE database. A TEAD4-binding site was observed ∼10 kb upstream of the transcriptional start site of human *PROX1* regulatory elements. (D) The DNA recognition motif of TEAD4. (E) PROX1 regulatory elements from the indicated mammals were aligned using Clustal Omega. A highly conserved GATA2-binding site reported by Kazenwadel et al. (2015) is within the red box. TEAD4-binding sites that are conserved either between rat/mouse or between dog/human/chimpanzee are underlined. Asterisks indicate conserved nucleotides. (F) ChIP followed by PCR using primers that flank the TEAD4-binding site revealed that YAP and TAZ directly associate with this regulatory element. *n*=3 western blot, qRT-PCR and ChIP assays. **P<0.01, ***P<0.001. Data are mean±s.e.m. Scale bar: 100 µm in E.

PROX1 is the master regulator of lymphatic vascular development and is required for the formation and maintenance of LVs and LVVs (Geng et al., 2016; Johnson et al., 2008; Srinivasan and Oliver, 2011). We verified that VP treatment of HLECs reduces PROX1 expression at the RNA and protein levels (Fig. 5A). Importantly, siRNA-mediated knockdown of YAP and TAZ also reduced PROX1 levels in HLECs (Fig. 5A,B).

The ENCODE database predicted a TEAD4-binding site in the regulatory elements of *PROX1*, close to the GATA2-binding site that was reported previously (Fig. 5C) (ENCODE Project Consortium, 2012; Kazenwadel et al., 2015). Using a targeted approach, we verified that a TEAD4-binding site (Fig. 5D) is indeed present in the upstream regulatory elements of *PROX1*, and is conserved among several mammals (Fig. 5E). Furthermore, ChIP-PCR revealed that YAP binds to this site in HLECs (Fig. 5F). Together, these data suggest that YAP/TAZ cooperates with TEAD4 to directly activate PROX1 expression in HLECs. However, the functional significance of this binding site is currently unknown, and it is likely that YAP and TAZ associate with multiple sites in the distal regulatory elements of genes, as described previously (Galli et al., 2015; Stein et al., 2015).

Activation of the Hippo signaling pathway leads to MST1/2-mediated phosphorylation and activation of LATS1/2, which in turn phosphorylate and inactivate YAP/TAZ. Therefore, to enhance YAP/TAZ activity in HLECs, we treated cells with XMU-MP-1, a chemical inhibitor of MST1 and MST2, or knocked down LATS1 and LATS2 (siLATS1/2). Both treatments resulted in the upregulation of the YAP and TAZ target genes *CTGF* and *CYR61*, as anticipated (Fig. S4A,B). In contrast, *PROX1* expression was downregulated by siLATS1/2 and XMU-MP-1 treatments (Fig. S4A,B), consistent with recent findings of Cho et al. (Cho et al., 2019). Thus, PROX1 expression in HLECs is delicately dependent on the activity of YAP and TAZ.

**Fig. 6.**
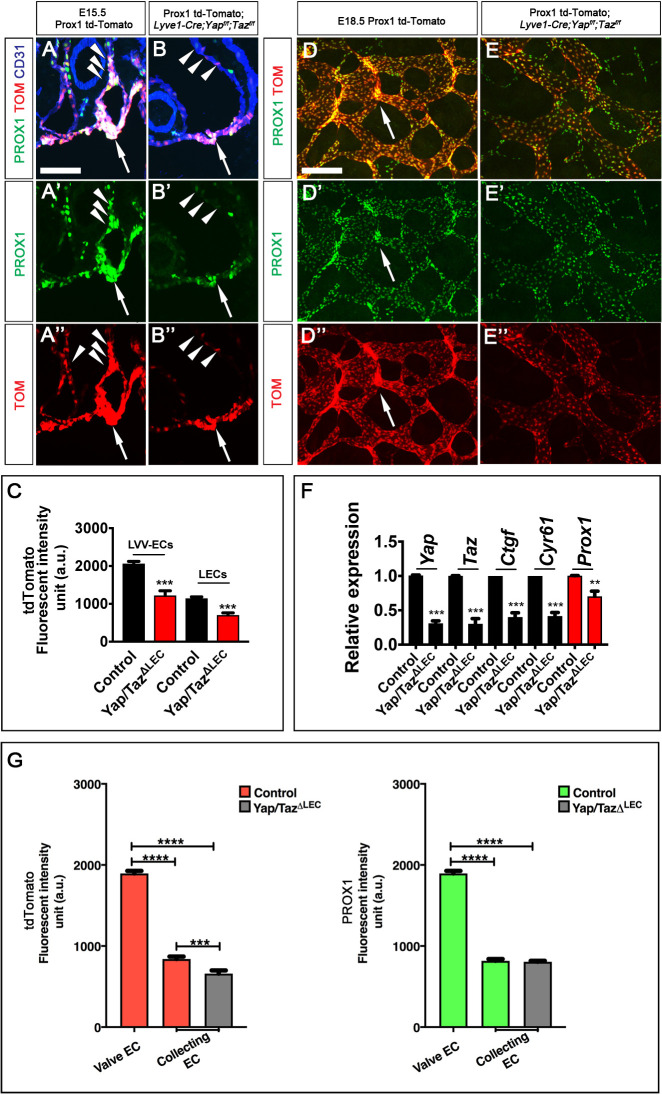
**YAP and TAZ regulate PROX1 expression in valvular endothelial cells.** (A-C) E15.5 Prox1-tdTomato (A-A″) and Prox1-tdTomato; *Lyve1-Cre;Yap^f/f^;Taz^f/f^* (B-B″) embryos were frontally sectioned and the expression of PROX1 and tdTomato (TOM) were analyzed by immunohistochemistry. The fluorescent intensity of tdTomato is semi-quantitatively measured in C. The expression of PROX1 and tdTomato were downregulated in LECs (arrowheads) and LVV-ECs (arrows) of embryos lacking YAP and TAZ. (D-E″) PROX1^high^;tdTomato^high^ LVs that were observed in E18.5 Prox1-tdTomato embryos (D-D″, arrows) were absent from Prox1-tdTomato; *Lyve1-Cre;Yap^f/f^;Taz^f/f^* littermates (E-E″). (F) LECs were sorted from the skin of E18.5 control or *Lyve1-Cre;Yap^f/f^;Taz^f/f^* littermates and qRT-PCR was performed using the extracted RNA. The expression of *Yap*, *Taz*, *Prox1*, and other YAP and TAZ target genes were downregulated in the LECs from mutant embryos. (G) The fluorescent intensities of PROX1 and tdTomato expression from samples shown in D,E were measured in a semi-quantitative manner. tdTomato expression was modestly downregulated in the lymphatic vessels of mutants. However, PROX1 expression appeared to be unchanged. In A-C, *n*=4 embryos and 8 pairs of LVVs for each genotype. In D-G, *n*=4 embryos per genotype. **P<0.01, ***P<0.001, ****P<0.0001. Data are mean±s.e.m. Scale bars: 100 µm in A-B″,D-E″.

To test whether YAP and TAZ regulate *Prox1* expression *in vivo*, we bred Prox1-tdTomato transgenic reporter mice into the *Yap/Taz^LECKO^* background. In the Prox1-tdTomato transgenic mice the expression of tdTomato is driven by ∼100 kb regulatory elements of *Prox1* (Gong et al., 2003). Thus, the transcriptional regulation of *Prox1* could be visualized using Prox1-tdTomato mice. Compared with Prox1-tdTomato littermate controls, Prox1-tdTomato; *Yap/Taz^LECKO^* embryos showed dramatically reduced expression of PROX1 and tdTomato in LVV-ECs (Fig. 6A,B, arrows; Fig. 6C) and in LECs of lymph sacs (Fig. 6A,B, arrowheads; Fig. 6C) at E15.5.

As mentioned previously, PROX1^high^; tdTomato^high^ LVs that were observed in the dermal lymphatic vessels of E18.5 Prox1-td-Tomato embryos (Fig. 6D, arrows) were absent in Prox1-td-Tomato; *Yap/Taz^LECKO^* littermates (Fig. 6E). Moreover, *Prox1* and other YAP/TAZ target genes showed reduced expression in LECs isolated from *Yap/Taz^LECKO^* embryos compared with controls (Fig. 6F). We wanted to determine whether the observed downregulation of *Prox1* in *Yap/Taz^LECKO^* embryos is due to the absence of LVs or also due to reduced *Prox1* expression in LECs. Therefore, we measured the intensities of PROX1 and tdTomato signals in a semi-quantitative manner from E18.5 Prox1-td-Tomato and Prox1-td-Tomato; *Yap/Taz^LECKO^* embryos (Fig. 6G). Expression of tdTomato, but not PROX1, was modestly downregulated in the LECs of Prox1-td-Tomato; *Yap/Taz^LECKO^* embryos. These results suggest that YAP and TAZ are necessary to maintain PROX1 expression primarily in valvular endothelial cells. This conclusion is supported by the fact that CTGF is expressed exclusively in the valves (Fig. 1, Fig. S1 and Sabine et al., 2015). In summary, we infer that *Yap* and *Taz* are thus required to maintain valvular endothelial cell identity at least in part by promoting *Prox1* expression.

### *Yap* and *Taz* genetically interact with *Prox1* in lymphatic vasculature development

**Fig. 7.**
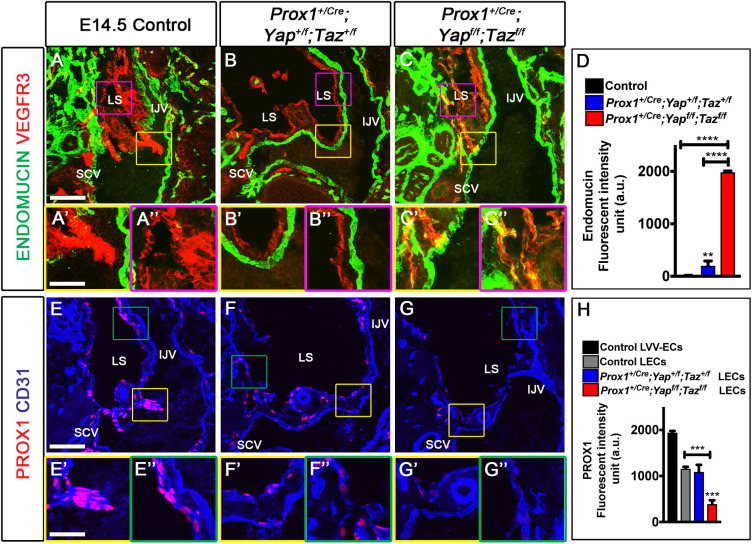
**Deletion of YAP and TAZ aggravates the lymphatic vascular defects associated with *Prox1* heterozygosity by further downregulating the expression of PROX1.** E14.5 wild-type (A-A″,E-E″), *Prox1^+/Cre^;Yap^+/f^;Taz^+/f^* (B-B″,F-F″) and *Prox1^+/Cre^;Yap/Taz^LECKO^* (C-C″,G-G″) littermates were sectioned and the expressions of endomucin and PROX1 were analyzed. (A-D) In wild-type and *Prox1^+/Cre^;Yap^+/f^;Taz^+/f^* embryos (A-B″), endomucin was expressed in the venous endothelial cells (A′,B′), but not in the LECs lining the lymph sacs (A″,B″). In contrast, patchy expression of endomucin was observed within the LECs of *Prox1^+/Cre^;Yap/Taz^LECKO^* embryos (C″). The fluorescent intensity of endomucin expression in LECs was measured and is plotted in D. (E-H) PROX1 was strongly expressed in the LVV-ECs of wild-type embryos (E′). LVVs were absent in *Prox1^+/Cre^;Yap^+/f^;Taz^+/f^* and *Prox1^+/Cre^;Yap/Taz^LECKO^* embryos (F′,G′). Additionally, PROX1 expression was downregulated in the LECs of *Prox1^+/Cre^;Yap/Taz^LECKO^* embryos (G″) compared with wild-type and *Prox1^+/Cre^;Yap^+/f^;Taz^+/f^* littermates (E″,F″). The fluorescent intensity of PROX1 expression was measured and is plotted in H. *n*=4 for each genotype. **P<0.01, ***P<0.001, ****P<0.0001. Data are mean±s.e.m. Scale bars: 200 µm in A,B,C,E,F,G; 100 µm in A′,A″,B′,B″,C′,C″,E′,E″,F′,F″,G′,G″.

To further investigate the relationship between PROX1, YAP and TAZ, we deleted *Yap* and *Taz* in PROX1-expressing cells, including LECs, using *Prox1^+/Cre^*. In these mice, one functional allele of *Prox1* is replaced by Cre recombinase, resulting in impaired LVV development (Srinivasan et al., 2010; Srinivasan and Oliver, 2011). E14.5 *Prox1^+/Cre^;Yap^+/f^;Taz^+/f^* embryos lacked LVVs, as expected, as did E14.5 *Prox1^+/Cre^;Yap/Taz^LECKO^* embryos (Fig. 7A-C). The glycoprotein endomucin is expressed in venous endothelial cells, but not in LECs (D'Amico et al., 2009). Both E14.5 wild-type and *Prox1^+/Cre^;Yap^+/f^;Taz^+/f^* embryos displayed endomucin expression in venous endothelial cells but not in LECs lining the lymph sacs (Fig. 7A,B). In contrast, E14.5 *Prox1^+/Cre^;Yap/Taz^LECKO^* embryos expressed endomucin in the lymph sacs (Fig. 7C,C″,D). This observation suggests that a subset of LECs in *Prox1^+/Cre^;Yap/Taz^LECKO^* embryos have abnormally acquired a partial blood vascular endothelial cell identity. This phenotype is also observed in embryos that completely lose *Prox1* after the specification of LECs (Johnson et al., 2008). Hence, we studied the expression of PROX1 by immunohistochemistry and found that it was indeed downregulated in the LECs of *Prox1^+/Cre^;Yap/Taz^LECKO^* embryos compared with their littermates (Fig. 7E-H).

We were unable to obtain *Prox1^+/Cre^;Yap/Taz^LECKO^* embryos beyond E14.5, likely due to the expression of Cre in hepatocytes, which rely on YAP and TAZ for their growth (Camargo et al., 2007; Dong et al., 2007). Therefore, we analyzed E17.5 *Prox1^+/Cre^;Taz^LECHet^;Yap^LECKO^* and *Prox1^+/Cre^;Yap^LECHet^;Taz^LECHet^* embryos. At E17.5, the lymphatic vessels of wild-type embryos had crossed the dorsal midline and LVs were observed at the lateral edges of the skin (Fig. S5A and data not shown). In contrast, the lymphatic vessels were dilated and did not reach the midline in *Prox1^+/Cre^;Taz^LECHet^;Yap^LECKO^* and *Prox1^+/Cre^;Yap^LECHet^;Taz^LECHet^* embryos (Fig. S5B,C). Intriguingly, *Prox1^+/Cre^;Taz^LECHet^;Yap^LECKO^* embryos developed cystic structures in their lymphatic vessels (Fig. S5C), whereas wild-type and *Prox1^+/Cre^;Yap^LECHet^;Taz^LECHet^* embryos did not. We propose that *Prox1^+/Cre^;Taz^LECHet^;Yap^LECKO^* embryos express relatively low levels of PROX1 compared with *Prox1^+/Cre^;Taz^LECHet^;Yap^LECHet^* embryos, which exacerbates defects in lymphatic vessel morphogenesis. Overall, our data reveal that genetic reduction of *Prox1* together with *Yap* and *Taz* results in the partial loss of LEC identity and improper morphogenesis of lymphatic vessels.

### VEGF-C signaling promotes PROX1 expression in HLECs through YAP and TAZ

PROX1 expression is positively regulated by VEGF-C signaling in HLECs (Koltowska et al., 2015; Srinivasan et al., 2014). We hypothesized that VEGF-C/VEGFR3 signaling activates PROX1 expression in LECs through YAP/TAZ. We treated high confluent (∼100%) or low confluent (∼50%) HLECs with 100 ng/ml VEGF-C and investigated the phosphorylation of YAP. We determined that pYAP is strikingly reduced by VEGF-C specifically in low confluent cells (Fig. S6). Thus VEGF-C regulates the phosphorylation of YAP in a cell density-dependent manner.

**Fig. 8.**
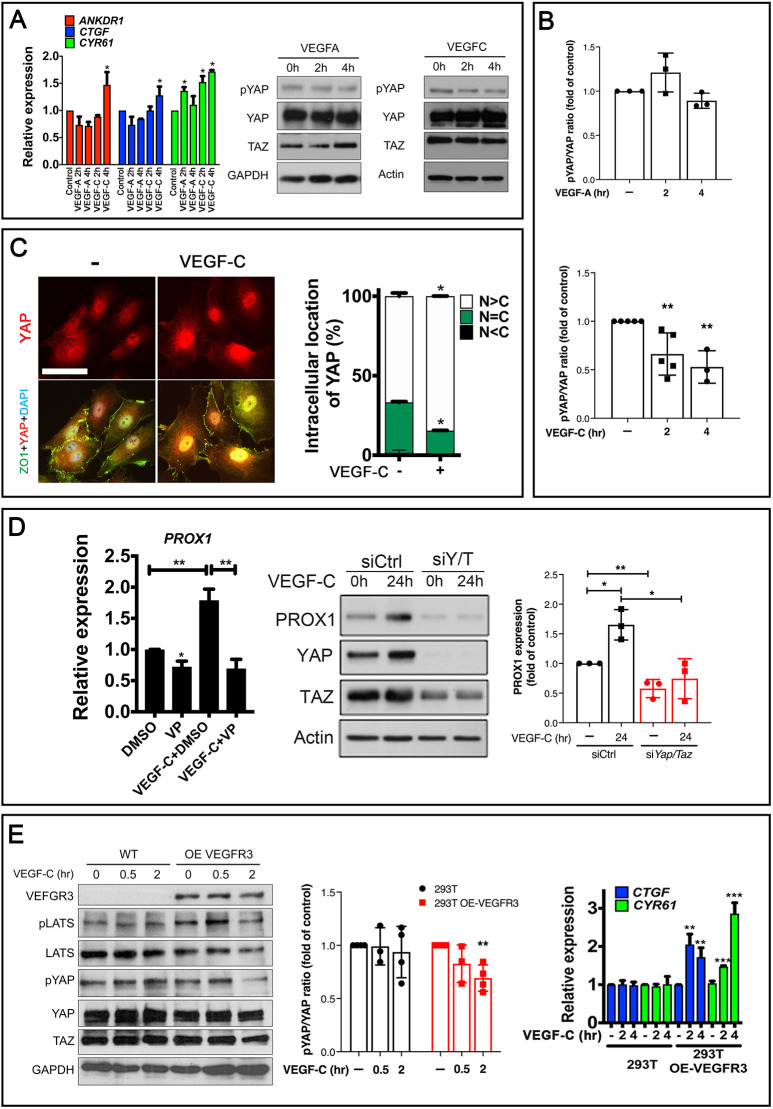
**VEGF-C enhances YAP and TAZ activity in HLECs and in 293T cells expressing VEGFR3.** (A,B) HLECs were treated with 100 ng/ml of VEGF-A or VEGF-C for 2 or 4 h following which RNA or protein was extracted to quantify the transcriptional activity of YAP and TAZ. Intensities of western bands were measured and plotted in B. The expression of YAP and TAZ target genes CTGF and CYR61 was upregulated by VEGF-C, but not by VEGF-A. Additionally, phosphorylation of YAP was specifically reduced by VEGF-C. (C) VEGF-C promoted the nuclear localization of YAP in HLECs. N, nuclear localization; C, cytoplasmic localization. (D) Treatment of HLECs with VEGF-C for 24 h enhanced the expression of PROX1 at both RNA (graph on the left) and protein (western blot on the right) levels. Inhibition of YAP and TAZ activity either by VP or by siRNA abolished the enhancement of PROX1 expression by VEGF-C. (E) 293T cells were stably infected with GFP or VEGFR3-overexpressing (OE) lentiviral particles and treated with VEGF-C for the indicated time points. Phosphorylation of YAP was reduced and the expression of YAP and TAZ target genes was increased by VEGF-C in VEGFR3 OE cells. *n*=5 for VEGF-C treatment in A; *n*=4 for western blotting in E; *n*=3 per each of the other experiments. *P<0.05, **P<0.01, ***P<0.001. Western blots were quantified and results presented as mean±s.d. Other data are presented as mean±s.e.m. Scale bar: 100 µm.

To test whether VEGF-C regulates Hippo signaling, we treated 50% confluent HLECs with VEGF-C and isolated RNA and protein. We found that the expression of YAP and TAZ target genes was significantly upregulated in VEGF-C-treated HLECs relative to controls (Fig. 8A). Additionally, VEGF-C treatment inhibited the phosphorylation of YAP, as mentioned above (Fig. 8A,B). In contrast, VEGF-A did not increase the expression of YAP and TAZ target genes or reduce the level of pYAP (Fig. 8A,B). Thus, YAP and TAZ activity is enhanced by VEGF-C, but not by VEGF-A in HLECs.

VEGF-C increased the levels of YAP in the nucleus of HLECs (Fig. 8C). Moreover, VP treatment and siRNA-mediated knockdown of YAP and TAZ inhibited the VEGF-C dependent upregulation of PROX1 in HLECs (Fig. 8D). We used 293T cells as a heterologous system to further verify whether VEGF-C and VEGFR3 signaling could enhance YAP and TAZ activity. We infected 293T cells with lentiviruses expressing VEGFR3. Following antibiotic selection, we treated stably infected cells with VEGF-C for 0.5 or 2 h. Western blotting and qRT-PCR confirmed that VEGF-C could indeed downregulate pYAP and upregulate the expression of YAP/TAZ target genes (Fig. 8E). Together, these data suggest that VEGF-C activates YAP and TAZ activity in HLECs and VEGFR3-expressing 293T cells. Furthermore, VEGF-C promotes PROX1 expression in HLECs through YAP and TAZ.

Next, we investigated whether the Hippo pathway regulates VEGF-C induced behaviors in HLECs. To examine HLEC migration, we performed an *in vitro* wound-healing assay. VEGF-C treatment promoted the migration of HLECs, as anticipated (Fig. S7A,B,E). Importantly, VP treatment significantly inhibited VEGF-C-induced HLEC migration (Fig. S7C-E). Additionally, siRNA-mediated depletion of YAP and TAZ significantly reduced VEGF-C-induced HLEC proliferation (Fig. S7F-J). Thus, YAP and TAZ are necessary for the proper response of HLECs to VEGF-C.

### VEGF-C promotes YAP and TAZ activity *in vivo*

Our *in vitro* studies indicate that VEGF-C signaling enhances YAP and TAZ activity. Our *in vivo* results have revealed that YAP and TAZ are required for lymphatic vessel, LVV, LV and venous valve morphogenesis. VEGF-C signaling is necessary for the development and maintenance of lymphatic vessels (Karkkainen et al., 2004, 2001; Nurmi et al., 2015). In addition, VEGFR3 is strongly expressed in LVs (Bazigou et al., 2009; Norrmén et al., 2009). Time-specific global deletion of *Vegfc* at E14.5 prevented the maturation of collecting lymphatic vessels and the development of LVs within the collecting lymphatic vessels (Nurmi et al., 2015). However, as the lymphatic vessels of these mice were thinner and immature, whether VEGF-C directly or indirectly regulates LV development remains unknown. VEGFR3 is also modestly expressed in LVVs and venous valves (Bazigou et al., 2011; Geng et al., 2016). Saphenous venous reflux is observed in individuals with Milroy's disease carrying mutations in *VEGFR3* (Mellor et al., 2010). Yet, it is unclear whether LVV and venous valve development requires VEGF-C and VEGFR3 signaling.

To investigate the relationship between VEGF-C and VEGF3 signaling and Hippo signaling *in vivo*, we analyzed three mouse models in which VEGF-C signaling was inhibited. We generated *Vegfc^+/CreERT2^* mice in which we replaced the open reading frame of *Vegfc* with cDNA coding for CreERT2. *Vegfc*^*+/CreERT2*^ mice recapitulated the phenotypes of *Vegfc*^*+/−*^ mice, including severe lymphatic vascular hypoplasia in the skin (Fig. S8). In addition, *Vegfc*^*+/CreERT2*^ mice developed chylous ascites at birth, swollen paws and tail and lymphatic vascular hypoplasia of the heart and diaphragm as reported previously in *Vegfc*^*+/−*^ mice (Karkkainen et al., 2004). *Vegfc^+/CreERT2^* mice will henceforth be referred to as *Vegfc^+/−^* mice in this study.

LVVs appeared normal in E15.5 *Vegfc^+/−^* embryos (Fig. S9A,B, arrows). Venous valves were also present in E17.5 *Vegfc^+/−^* embryos (Fig. S10A,B, red arrowheads). Similarly, LVVs and venous valves were observed in *Vegfr3^+/chy^* embryos, which express a dominant-negative allele of VEGFR3 and phenocopy *Vegfc^+/−^* mice (Fig. S9C,D, pseudo-colored in magenta and yellow, respectively) (Karkkainen et al., 2001). These results suggest that, despite severe lymphatic vascular defects, LVV and venous valve development are not affected in *Vegfc^+/−^* and *Vegfr3^+/chy^* embryos.

**Fig. 9.**
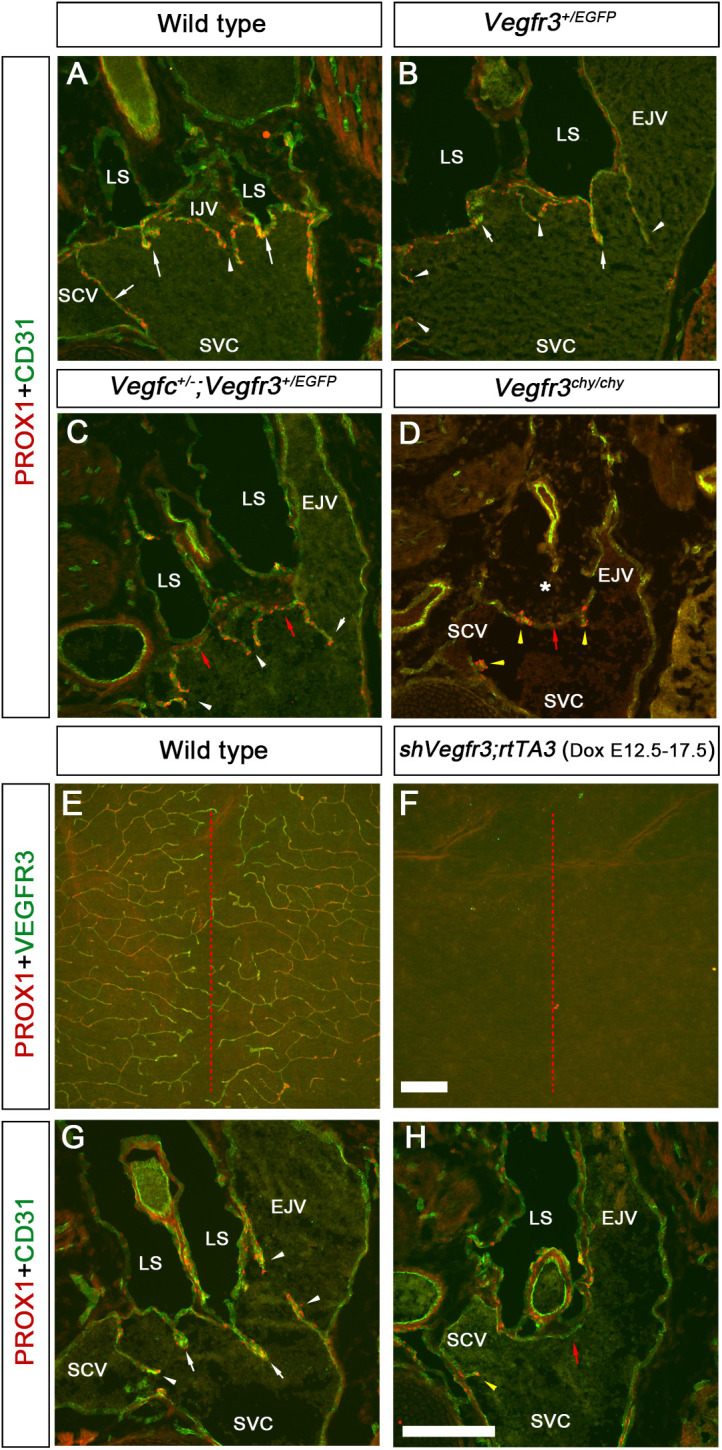
**VEGF-C regulates the formation of LVVs.** (A-D) LVVs (arrows) and venous valves (arrowheads) were found in E17.5 wild-type (A) and *Vegfr3^+/EGFP^* (B) embryos. (C) *Vegfr3^+/EGFP^;Vegfc^+/−^* embryos had the venous valves (arrowheads). However, LVVs were absent in the areas where they would have normally formed (red arrows). (D) E17.5 *Vegfr3^chy/chy^* embryos lacked lymph sacs (asterisk) and LVVs (red arrow). In addition, only a few venous valve-forming cells were observed (yellow arrowheads). (E,F) Lymphatic vessels were absent from the dorsal skin of E17.5 *shVegfr3;rtTA3* embryos that were treated with doxycycline from E12.5 (F). (G,H) Lymph sacs (LS), LVVs (G, arrows) and venous valves (G, arrowheads) that were seen in E17.5 wild-type embryos were absent from *shVegfr3;rtTA3* littermates. Red arrow in H indicates the location at which LVVs would have normally formed. Yellow arrowhead in H indicates the few remaining venous valve-forming endothelial cells. LS, lymph sac; IJV, internal jugular vein; EJV, external jugular vein; SCV, subclavian vein; SVC, superior vena cava. In A-D,G,H, *n*=3 embryos per genotype and 2 LVV/venous valve complexes per embryo; in E,F, *n*=5 dorsal skins from 5 embryos per genotype. Scale bars: 200 µm in A-D,G,H; 1000 µm in E,F.

We analyzed LVVs and venous valves in E17.5 *Vegfr3^+/EGFP^;Vegfc^+/−^* embryos in which VEGF-C and VEGFR3 signaling is expected to be more than in *Vegfc^−/−^*, but less than in *Vegfc^+/−^* embryos. LVVs and venous valves were observed in wild-type and *Vegfr3^+/EGFP^* embryos, as anticipated (Fig. 9A,B, white arrows and white arrowheads, respectively). Lymph sacs were observed, but LVVs were absent in E17.5 *Vegfr3^+/EGFP^;Vegfc^+/−^* embryos (Fig. 9C, red arrows). In contrast, venous valves of *Vegfr3^+/EGFP^;Vegfc^+/−^* embryos appeared indistinguishable from control littermates (Fig. 9C, white arrowheads). These results suggest that LVVs are more reliant on VEGF-C signaling for their development compared with venous valves.

We also analyzed E17.5 *Vegfr3^chy/chy^* embryos in which VEGF-C/VEGFR3 signaling is completely abolished (Karkkainen et al., 2001; Zhang et al., 2010). Lymph sacs and LVVs were absent from these embryos (Fig. 9D, asterisk and red arrow, respectively). Importantly, very few PROX1^+^ cells were observed on the walls of veins where venous valves normally form (Fig. 9D, yellow arrowheads). It is possible that the lack of lymph sacs resulted in the absence of LVVs and venous valves. To test this possibility, we generated a new double-transgenic mouse model to abolish VEGF-C and VEGFR3 signaling in a time-specific manner after the development of lymph sacs. In these mice, 3rd generation reverse tetracycline-regulated transactivator (rtTA3) is expressed from the *Rosa26* locus and is regulated by CAGG regulatory elements (CMV enhancer with chicken β-actin promoter). In addition, a miRNA-based shRNA targeting *Vegfr3* is expressed from tetracycline response element (TetO) that is knocked into the constitutively active *Col1a1* locus (Premsrirut et al., 2011). Doxycycline (Dox), when administered in food, will activate rtTA3. Transcriptionally active rtTA3 will bind TetO to activate the expression of shRNA that will knock down *Vegfr3*. We exposed these *ShVegfr3;rtTA3* embryos to Dox from E12.5, after the formation of lymph sacs, and analyzed them at E17.5. The dorsal skin of E17.5 *ShVegfr3;rtTA3* embryos was devoid of lymphatic vessels, which is consistent with the phenotypes of *Vegfc^+/−^* and *Vegfr3^+/chy^* embryos (Fig. 9E,F) (Karkkainen et al., 2004, 2001). Lymph sacs were observed in E17.5 *ShVegfr3;rtTA3* embryos (Fig. 9G,H). However, they lacked LVVs (Fig. 9H, red arrow) and their venous valves were severely reduced in size (Fig. 9H, yellow arrowhead). The results from *Vegfr3^chy/chy^* and *ShVegfr3;rtTA3* embryos indicates that VEGF-C signaling is essential for the development of LVVs and venous valves.

**Fig. 10.**
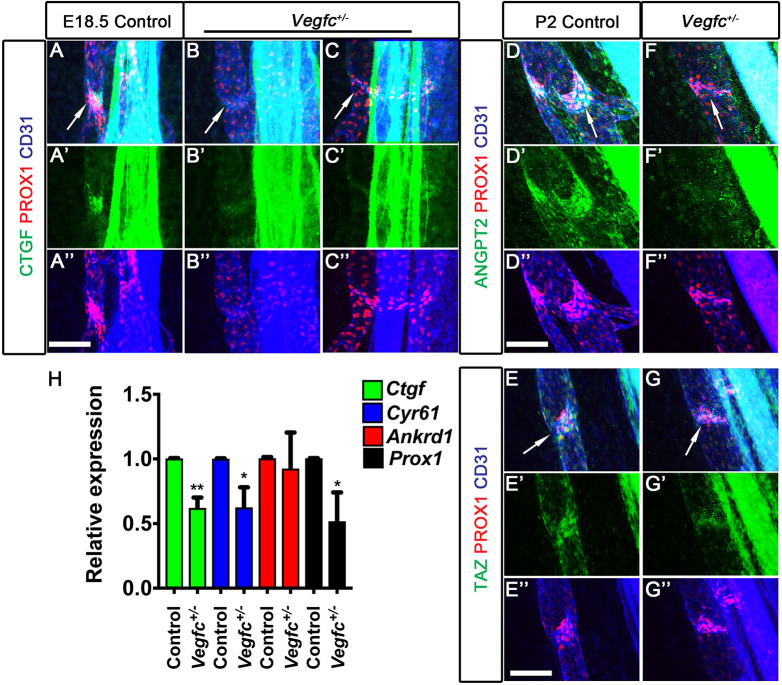
**Expression of YAP and TAZ targets is downregulated in the LVs of *Vegfc^+/−^* embryos.** (A-G″) LVs in the mesenteric lymphatic vessels of E18.5 (A-C″) or P2 (D-G″) wild-type and *Vegfc^+/−^* embryos were analyzed for the expression of PROX1 and other YAP/TAZ target genes. PROX1 (A,A″,D,D″,F,F″), CTGF (A,A′), ANGPT2 (D,D′) and TAZ (F,F′) were strongly expressed in the LVs of wild-type embryos and pups. In contrast, the LVs of *Vegfc^+/−^* mice appeared immature, as interpreted from the lack of PROX1^+^ clusters at sites where LVs would have normally formed (B, arrow), the dispersed characteristic of PROX1^+^ cells in the LVs that are forming (C, arrow, and C″), the absence of the dome-shaped structure (F, arrow) and reduced expressions of CTGF (B′,C′), ANGPT2 (F′) and TAZ (G′). (H) LECs were sorted from the mesentery of P2 wild-type and *Vegfc^+/−^* pups and qRT-PCR was performed. *Prox1* and other YAP/TAZ target genes were downregulated in the LECs of *Vegfc^+/−^* pups. *n*=4 for each genotype. *P<0.05, **P<0.01. Data are mean±s.e.m. Scale bars: 200 µm in A-G″.

Having determined that VEGF-C signaling is necessary for valve development, we tested whether YAP/TAZ activity is downregulated in *Vegfc^+/−^* embryos. The LV-ECs of E18.5 wild-type embryos had aggregated with each other, giving the LVs a compact and mature appearance (Fig. 10A, arrow). The YAP/TAZ target protein CTGF was expressed in the LVs of wild-type embryos (Fig. 10A, arrow). In contrast, there were fewer LVs in E18.5 *Vegfc^+/−^* embryos (Fig. 10B; data not shown). The LVs that were observed in E18.5 *Vegfc^+/−^* embryos were immature and CTGF was dramatically downregulated in the developing LVs of *Vegfc^+/−^* littermates (Fig. 10C, arrow). LVs further matured in 2-day-old (P2) wild-type pups, and expressed TAZ and the YAP and TAZ target protein ANGPT2 (Fig. 10D,E, arrows). In contrast, the LVs of P2 *Vegfc^+/−^* littermates remained immature and did not express ANGPT2 (Fig. 10F, arrow). TAZ expression was also downregulated in the LVs of P2 *Vegfc^+/−^* pups (Fig. 10G, arrow). YAP and TAZ expression was also downregulated in the mesenteric LVs of P2 *Vegfr3^+/chy^* pups and E18.5 *shVegfr3;rtTA3* embryos that were exposed to Dox for 2 days (Fig. S11). We sorted LECs from the mesentery of P2 wild-type and *Vegfc^+/−^* pups, extracted RNA and performed qRT-PCR to estimate the expression of YAP and TAZ target genes. The expression of *Prox1* and other YAP and TAZ target genes, *Ctgf* and *Cyr61*, was downregulated in the LECs of *Vegfc^+/−^* pups (Fig. 10H). These results suggest that VEGF-C enhances YAP and TAZ activity in LVs.

In summary, VEGF-C and VEGFR3 signaling regulates LV, LVV and venous valve development. YAP and TAZ are essential mediators of VEGF-C signaling during valve development. VEGF-C signaling enhances the transcriptional activity of YAP and TAZ to promote the expression of genes such as *PROX1*, *CTGF* and *ANGPT2* that are potently expressed in valves. Consequently, in the absence of YAP and TAZ, the development of lymphatic vessels, LV, LVV and venous valves is defective.

## DISCUSSION

Our work has identified several previously unknown mechanisms that operate during lymphatic vascular development. We and others have previously reported a feedback loop between PROX1 and the VEGF-C/VEGFR3 signaling pathway in LECs (Koltowska et al., 2015; Srinivasan et al., 2014). PROX1 directly activates the expression of VEGFR3. In turn, VEGF-C and VEGFR3 signaling maintains the expression of PROX1. The mechanisms by which VEGF-C and VEGFR3 signaling regulates PROX1 expression are not fully understood. Here, we have identified YAP and TAZ as critical mediators of the PROX1 expression in response to VEGF-C signaling. VEGF-C is unable to enhance PROX1 expression in HLECs in the absence of YAP and TAZ. This finding establishes YAP and TAZ as crucial components of the PROX1 and VEGFR3 feedback loop.

PROX1, FOXC2, GATA2 and VEGFR3 are expressed at much higher levels in valvular endothelial cells compared with the rest of the lymphatic vasculature (Bazigou et al., 2009; Geng et al., 2016; Kazenwadel et al., 2015; Norrmén et al., 2009). Previous reports, including our own, have shown that PROX1, FOXC2 and GATA2 are necessary for the development of LVs and LVVs (Geng et al., 2016; Kazenwadel et al., 2015; Mahamud et al., 2019; Norrmén et al., 2009; Petrova et al., 2004). However, whether VEGFR3 is necessary for the development of valves has not been demonstrated, although superficial venous valve reflex is observed in individuals with Milroy's disease, which is characterized by heterozygous inactivating mutations in *VEGFR3* (Mellor et al., 2010). In this work we have shown that VEGF-C and VEGFR3 signaling is indeed required for the proper development of LVs, LVVs and venous valves. Furthermore, we show that VEGF-C and VEGFR3 signaling activates YAP and TAZ, which in turn maintains the expression of PROX1 in valvular endothelial cells. These data suggest that the PROX1 and VEGFR3 feedback loop is operational in developing valves. Intriguingly, deletion of *Yap* and *Taz* did not affect the differentiation of valvular endothelial cells. Instead, between E16.5 and E18.5, PROX1 expression was downregulated and LVs and LVVs degenerated in the absence of YAP and TAZ. Thus, YAP and TAZ are crucial for the PROX1 and VEGFR3 feedback loop during the maturation stage of valve development.

YAP and TAZ are necessary for the proper functioning of migratory tip cells during angiogenesis (Kim et al., 2017; Neto et al., 2018; Sakabe et al., 2017; Wang et al., 2017). However, lymphangiogenesis happens normally in *Yap/Taz^LECKO^* embryos until E16.5, after which their lymphatic vessels become dramatically dilated. Considering the facts that CTGF expression is enriched in valves, and that the onset of lymphatic vessel defects in *Yap/Taz^LECKO^* embryos coincides with the degeneration of LVs and LVVs, we suggest that YAP and TAZ are playing a prominent role in valvular endothelial cells. Thus, degeneration of LVs and LVVs in *Yap/Taz^LECKO^* embryos prevents lymphatic drainage, resulting in the dilation of lymphatic vessels. Nevertheless, lymphatic vessels of *Prox1*-heterozygous embryos that lack three out of four *Yap/Taz* alleles develop abnormal cysts that are not seen in *Prox1^+/−^* embryos that lack LVVs and LVs. Thus, YAP and TAZ are likely also necessary in LECs. Additionally, deletion of *Yap* and *Taz* in a *Prox1*-heterozygous background resulted in the abnormal expression of endomucin in the LECs at E14.5. This phenotype is partially reminiscent of embryos in which *Prox1* was deleted after the specification of LECs (Johnson et al., 2008). These results suggest that YAP and TAZ play a modest role in promoting *Prox1* expression in LECs.

In contrast to our findings, Cho et al. showed that YAP and TAZ inhibit PROX1 expression in the developing lymphatic vessels (Cho et al., 2019). The differences could be due to the distinct Cre lines that were used. Although we used *Lyve1-Cre*, Cho et al. used Prox1-CreERT2 (Bazigou et al., 2011). Although Cho et al. observed severe edema in their mutant embryos, we did not observe edema in most of our samples. The more severe phenotype observed by Cho et al. could be due to more potent and/or rapid gene deletion by Prox1-CreERT2. As mentioned above, we suspect that YAP and TAZ play a primary role in maintaining the integrity of LVs and LVVs. Degeneration of LVVs at an earlier stage in the *Yap/Taz^LECKO^* embryos generated by Cho et al. might have affected lymphatic drainage earlier, which in turn would have prevented lymphatic vessel maturation, resulting in sustained expression of PROX1. Deleting *Yap* and *Taz* at various developmental time points and in specific compartments of the lymphatic vasculature (LEC progenitors, LECs, LVs, LVVs, tip cells and stalk cells) could test these possibilities and provide better resolution of YAP and TAZ activity.

There are also differences in *in vitro* data generated by Cho et al. and us. Whereas we have determined that inhibition of YAP and TAZ activity by VP or siRNA inhibits PROX1 expression, Cho et al. draw the opposite conclusion from their results. It is possible that these differences are due to differences in cell lines or culture conditions that were used. Nevertheless, there are important points of congruity between our findings and those of Cho et al. Specifically, we also found that overactivation of YAP and TAZ, via pharmacological inhibition of MST1/2 or RNAi-mediated depletion of LATS1/2 reduces PROX1 expression in confluent HLECs. These results – both loss and overactivation of YAP and TAZ could result in the downregulation of PROX1 expression in the lymphatic vasculature – suggest that a precise level of YAP and TAZ activity regulates PROX1 expression.

Cell-density appears to function as a buffer that regulates YAP and TAZ activity in response to VEGF-C. We found that VEGF-C could reduce the phosphorylation of YAP and promote the expression of YAP and TAZ target genes only in HLECs grown under low-cell density. These later results are consistent with the report by Grimm et al., who elegantly showed that YAP is necessary for VEGF-C-induced proliferation of LECs in zebrafish (Grimm et al., 2019). The mechanisms and significance of this finding is currently unknown.

Many important questions remain regarding the role of YAP and TAZ in lymphatic vascular development. Multiple signaling pathways including shear stress, Wnt/β-catenin signaling and integrin/extracellular matrix signaling regulate YAP/TAZ activity (Azzolin et al., 2014, 2012; Dupont et al., 2011; Frye et al., 2018; Sabine et al., 2015; Zheng and Pan, 2019). These signaling pathways also regulate lymphatic vascular development and valve morphogenesis (Cha et al., 2016, 2018; Frye et al., 2018; Sabine et al., 2012, 2015). It remains to be seen whether these signaling pathways could activate YAP and TAZ in a functionally relevant manner during lymphatic vascular development. In primary human LECs, the transcription factor FOXC2 inhibits abnormal activation of TAZ by oscillatory shear stress (Sabine et al., 2015). Consequently, deletion of FOXC2 from the LVs of postnatal mice results in the abnormal activation of TAZ leading to the loss of quiescence, increased proliferation and apoptosis (Sabine et al., 2015). Whether deletion of *Yap* and *Taz* could ameliorate valve defects in mice lacking *Foxc2* needs to be tested. Finally, XMU-MP-1 is used pharmacologically to protect the heart against pressure overload (Triastuti et al., 2019). It will be important to determine whether XMU-MP-1 could be repurposed to treat lymphedema when VEGFR3 signaling is compromised, as in the case of Milroy's disease.

## MATERIALS AND METHODS

### Antibodies

Primary antibodies for immunohistochemistry on mouse tissues were as follows: rabbit anti-PROX1 (11-002, Angiobio; 1:500), goat anti-human PROX1 (AF2727, R&D Systems; 1:500), sheep anti-mouse FOXC2 (AF6989, R&D Systems; 1:300), goat anti-mouse VEGRF3 (AF743, R&D Systems; 1:300), rat anti-mouse CD31 (553,370, BD Pharmingen; 1:500), goat anti-human ANGPT2 (AF623, R&D Systems; 1:300), rabbit anti-mouse LYVE-1 (11-034, Angiobio; 1:3000), rabbit anti-human/mouse anti-YAP/TAZ (8418, Cell Signaling; 1:200), rabbit anti-human/mouse TAZ (HPA007415, Sigma; 1:200), rabbit anti-human/mouse CTGF (ab6992, Abcam; 1:200), rat anti-mouse endomucin (14-5851, eBioscience; 1:3000), rabbit anti-ZO-1 (40-2200, Invitrogen; 1:100), rabbit anti-human/mouse pHH3 (06-570, Millipore; 1:300) and goat anti-mouse GATA2 (AF2046, R&D Systems; 1:300).

Primary antibodies for immunocytochemistry: mouse anti-mouse/human YAP (sc-101199, Santa Cruz; 1:100), mouse anti-mouse/human TAZ (560235, BD Pharmingen; 1:100), goat anti-human PROX1 (AF2727, R&D Systems; 1:500), rabbit anti-mouse/human ZO-1 (40-2200, Invitrogen; 1:100) and rabbit anti mouse/human β-catenin (9562, Cell Signaling; 1:200).

Secondary antibodies for immunohistochemistry and immunocytochemistry were as follows: Cy3-conjugated donkey anti-rabbit (711-165-152; 1:500), Cy3-conjugated donkey anti-sheep (711-165-147; 1:500), and Cy5-conjugated donkey anti-rat (712-175-150; 1:500) and Alexa 488-conjugated donkey anti-goat (705-545-147; 1:500) were purchased from Jackson ImmunoResearch Laboratories. Alexa 488-conjugated goat anti-chicken (A-11039; 1:500) and Alexa 488-conjugated donkey anti-rat (A-21208; 1:500) were purchased from Life Technologies.

Primary antibodies for western blotting were as follows: mouse anti- β-actin (A5441, Sigma; 1:100,000), goat anti-human PROX1 (AF2727, R&D Systems; 1:1000), mouse anti-mouse/human YAP (sc-101199, Santa Cruz; 1:1000), mouse anti-mouse/human TAZ (560235, BD Pharmingen; 1:2000), rabbit anti-human/mouse pYAP (4911, Cell Signaling; 1:500), rabbit anti human/mouse Lats1/2 (3477, Cell Signaling; 1:500), rabbit anti-human/mouse pLATS1/2 (8654, Cell Signaling; 1:500), rabbit anti human/mouse pERK1/2 (4376, Cell Signaling; 1:1000), rabbit anti human/mouse ERK1/2 (4695, Cell Signaling; 1:1000), mouse anti-human VEGFR3 (MAB3757, Millipore; 1:1000) and rabbit anti GAPDH (PAB13195, Abnova; 1:1000).

HRP-conjugated secondary antibodies for western blotting were as follows: goat anti-mouse IgG (A4416, Sigma; 1:5000), goat anti-rabbit IgG (GtxRb-003-EHRPX, Immuno Reagent; 1:5000), donkey anti-goat IgG (705-035-003, Jackson ImmunoResearch; 1:5000) and donkey anti-sheep IgG (HAF016, R&D Systems; 1:5000).

### Cells and chemicals

We used de-identified primary human lymphatic endothelial cells (HLECs) for experiments. Dr Donwong Choi (Keck School of Medicine, University of Southern California, USA) provided the HLECs (Choi et al., 2019, 2017a,b, 2016). HLECs were grown on gelatin-coated plates or glass slides and were maintained in EBM2 media from Lonza. All experiments were conducted using passage 5-6 cells. HLECs were treated as potential biohazards and were handled according to institutional biosafety regulations.

VEGF-C (9199-vc-025/CF, R&D Systems), verteporfin (SML0534, Sigma Aldrich) and XMU-MP-1 (S8334, Selleck Chemicals) were diluted in manufacturer's recommended solvents.

### Chromatin immunoprecipitation

ChIP assays were performed using the EZ-ChIP kit (MilliporeSigma) according to the manufacturer's instructions. Around 1.0×10^7^ HLECs were used per ChIP. Briefly, HLECs were grown on a culture dish at around 100% confluence. Subsequently, HLECs were fixed in 1% formaldehyde for 10 min at room temperature and glycine at a final concentration of 0.125 M was added for 5 min. Cells were washed with 20 ml of ice-cold PBS twice (10 min each) and harvested. Cells were lysed and sonicated as previously described (Cha et al., 2016, 2018).

Chromatin immunoprecipitation was performed using 0.2 μg of rabbit anti-human YAP (ab52771, Abcam) or 1.0 μg of normal rabbit IgG antibody (sc-2027, Santa Cruz Biotechnology). Following ChIP, PCR or q-PCR was performed using primers flanking the predicted TEAD4-binding site or a control site within the *PROX1* promoter (5′-AGCCAGGGAATGAGTACAGG-3′ and 5′-AGGAAGCCTGTGCATTAACAC-3′).

### Immunohistochemistry of tissues

Immunohistochemistry on sections was carried out according to our previously published protocols (Cha et al., 2016, 2018; Geng et al., 2016). Briefly, freshly collected embryos were washed in 1× PBS and fixed in 4% paraformaldehyde (PFA) overnight at 4°C. Subsequently, the embryos were washed three times (10 min each) in ice-cold PBS, incubated in 15% sucrose overnight at 4°C and then in 30% sucrose at 4°C until fully submerged in the solution. Embryos were then cryo-embedded in OCT solution (Sakura, Tokyo, Japan). Cryosections (12 μm) were prepared using a cryotome (Thermo Fisher Scientific, HM525 NX) and immunohistochemistry was performed using the indicated antibodies. E11.5 embryos were sectioned in a transverse orientation and E12.0-E16.5 embryos were sectioned frontally. Several consecutive sections were analyzed to determine the presence or absence of LVVs and VVs.

Whole-mount immunohistochemistry using embryonic skin or guts was performed according to our previous protocol (Cha et al., 2016, 2018). Either whole embryos or isolated guts were washed in 1×PBS and fixed in 1% PFA for 1 h to overnight (depending on the antibody) at 4°C. Subsequently, the dorsal skins were isolated, washed and samples were immunostained using the iDISCO protocol (Renier et al., 2014). Samples were visualized and analyzed as described previously (Cha et al., 2016, 2018). Fluorescent intensities were measured in a semi-quantitative manner using ImageJ.

### Immunostaining of cells

Cells were fixed in 1% PFA at room temperature for 30 min. Cells were subsequently permeabilized with 0.3% Triton X-100 for 10 min at room temperature, then washed with PBST (PBS+0.1% Triton-X100) and blocked in 0.5% BSA PBST for 1 h at room temperature. Samples were incubated with primary antibodies at 4°C overnight. Samples were then washed with PBST and incubated with secondary antibodies for 2 h at room temperature, and then washed with PBST three times (10 min each), mounted and visualized as previously described (Cha et al., 2016, 2018).

### Knockdown of YAP, TAZ, LATS1 and LATS2

HLECs were seeded at 40-50% confluence on plates. The following day, cells were transfected with equal amounts of siControl (51-01-14-03, Integrated DNA Technologies), siYAP (hs.Ri.YAP1.13.1, hs.Ri.YAP1.13.3) and siTAZ (hs.Ri.TAZ.13.2, hs.Ri.TAZ.13.3) or siLATS1 (hs.Ri.LATS1.13.1, hs.Ri.LATS1.13.3) and siLATS2 (hs.Ri.LATS2.13.2, hs.Ri.LATS2.13.3) using Lipofectamine RNAimax (Thermo Fisher Scientific) according to the manufacturer's instructions. After 2-3 days cells were treated with VEGF-C and harvested with Trizol (Invitrogen) or RIPA buffer for qRT-PCR or western blotting, respectively.

### Mice

*Prox1^+/Cre^* (Srinivasan et al., 2010), Tg(Prox1-tdTomato) (Gong et al., 2003), *Lyve1-Cre* (Pham et al., 2010), *Vegfr3^+/EGFP^* (Ichise et al., 2010), *Yap^flox^* and *Taz^flox^* mice have been described previously (Xin et al., 2013, 2011)*. Vegfc^+/CreERT2^* mice were generated by Cyagen by inserting the cDNA for CreERT2 immediately downstream of ATG at the *Vegfc* locus. Mirimus generated the *shVegfr3;rtTA3* mice according to their published protocols (Premsrirut et al., 2011).

*Prox1^+/Cre^* mice were maintained in NMRI background. Other mice were maintained in C57BL6 or C57BL6/NMRI mixed backgrounds. We used both male and female mice for the experiments. All mice were housed and handled according to the institutional IACUC protocols.

### Regulatory element analysis

*PROX1* regulatory elements were analyzed through ENCODE (ENCODE Project Consortium, 2012). For targeted analysis, *PROX1* regulatory element sequences obtained from Ensembl were aligned using Clustal Omega, and Homer was used for TEAD4 binding site identification (Heinz et al., 2010; Madeira et al., 2019; Yates et al., 2020).

### RNA-seq analysis

Total RNA was purified from HLECs treated with VP for 2 h. RNA was subjected to ribosomal RNA depletion followed by Truseq stranded total RNA library preparation according to the manufacturer's instruction (Illumina). The resulting RNA-seq libraries were analyzed on the Illumina HiSeq sequencing platform.

The obtained sequencing reads were mapped with the bowtie2 algorithm using the RefSeq annotations (hg19 genome build) (Langmead and Salzberg, 2012). We used the RNA-seq analysis work flow within the Partek Genomics Suite (Partek Incorporated) for quantitation and statistical analysis (ANOVA) of the transcriptome data. We identified those transcripts that exhibited statistically significant differential expression in the VP-treated samples compared with the control samples. We rank ordered the two lists based on the expression level and magnitude of change. Using these rank-ordered lists, we performed gene ontology (GO) analysis for enriched biological terms (Eden et al., 2009). The differentially expressed genes were analyzed using the functional annotation platform of DAVID (Huang et al., 2009a,b).

### Scanning electron microscopy

Scanning electron microscopy was performed according to our previous protocol (Geng et al., 2016; Geng and Srinivasan, 2018).

### Statistical analysis

For biochemical analysis, *n* indicates the number of times the experiments were independently performed and for histological analysis *n* indicates the number of embryos analyzed per genotype. All experiments were performed at least three times or more. Data were presented as mean±s.e.m. GraphPad Prism 7 software was used to perform the statistical analysis. Data were analyzed using the unpaired, two-tailed, Student's *t-*test. *P*<0.05 was considered significant.

### Western blot

Cells were harvested with RIPA lysis buffer and western blots were performed using standard protocol. The density of bands was measured by ImageJ and presented as mean±standard deviation (s.d.).

## Supplementary Material

Supplementary information

Reviewer comments
