# Supplementary material for: YAP and TAZ maintain PROX1 expression in the developing lymphatic and lymphovenous valves in response to VEGF-C signaling
Source: Development. 2020 Dec 13;147(23):dev195453. doi: 10.1242/dev.195453 (PMC7758626; doi:10.1242/dev.195453)
Supplement: Supplementary information [file develop-147-195453-s1.pdf]

Supplementary Figure 1

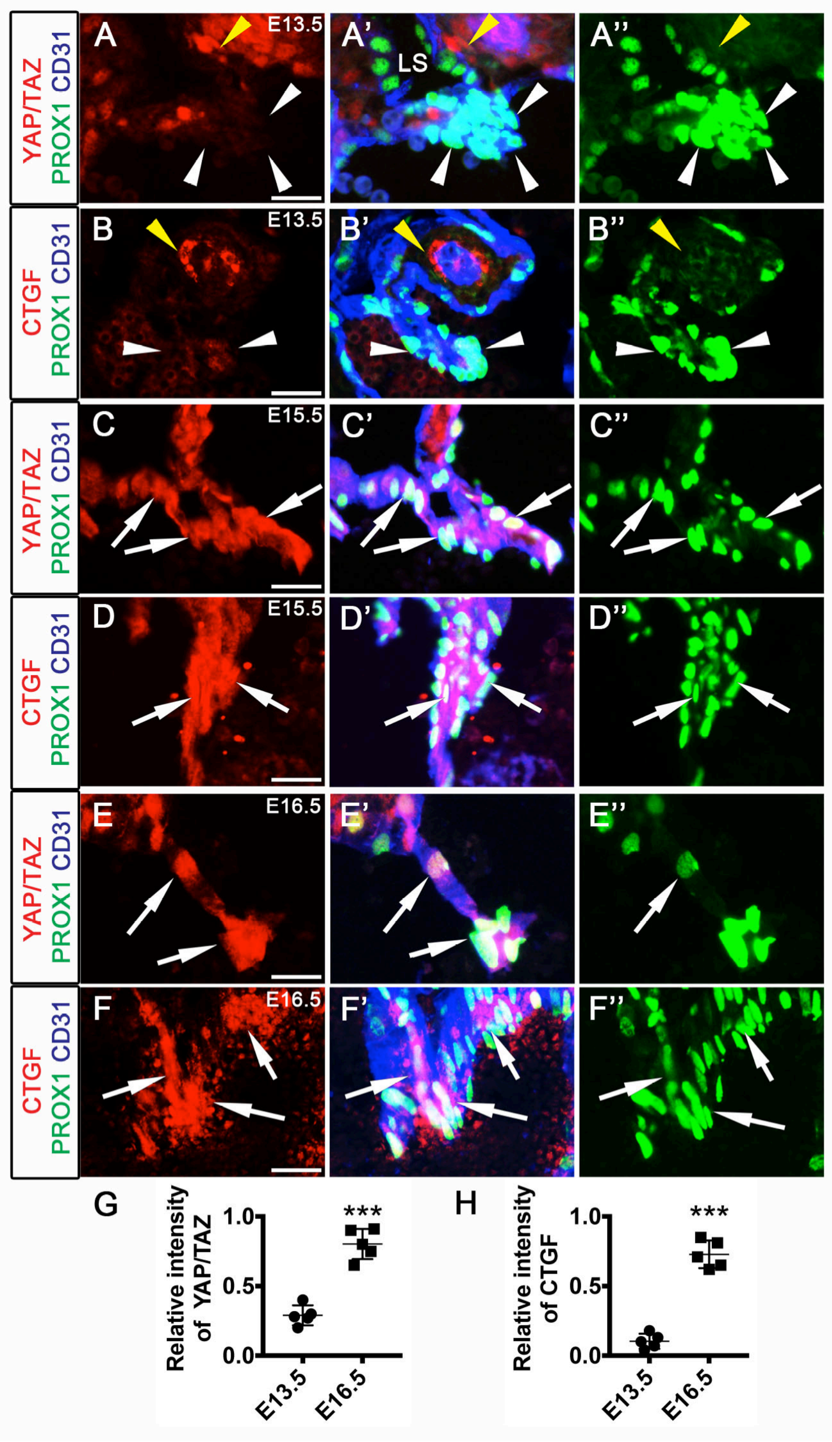

Figure S1: YAP/TAZ activity is upregulated in mature LVVs.

(A-F) LVVs of E13.5 (A, B), E15.5 (C, D) and E16.5 (E, F) mouse embryos were analyzed by IHC using the indicated antibodies.

(A, B) YAP/TAZ were not strongly expressed in the LVV-ECs (A, white arrowheads) but were strongly expressed in vascular smooth muscle cells around the nearby artery (A, yellow arrowhead) at E13.5. Correspondingly, YAP/TAZ target CTGF was not expressed in LVV-ECs (B, white arrowheads) at this stage. However, CTGF was expressed in the vascular smooth muscle cells surrounding the nearby artery (B, yellow arrowhead).

(C-F) Expressions of YAP/TAZ and CTGF were increased in the LVV-ECs (arrows) of E15.5 (C, D) and E16.5 (E, F) embryos.

(G, H) The fluorescent intensities of YAP/TAZ (G) and CTGF (H) were quantified in a semi-quantitative manner from E13.5 and E16.5 embryos and plotted.

Statistics: n = 4 for each experiment. *** $P < 0.001$. Data are presented as mean ± SEM.

Measuring bar: (A-F) 100 μm.

Supplementary Figure 2

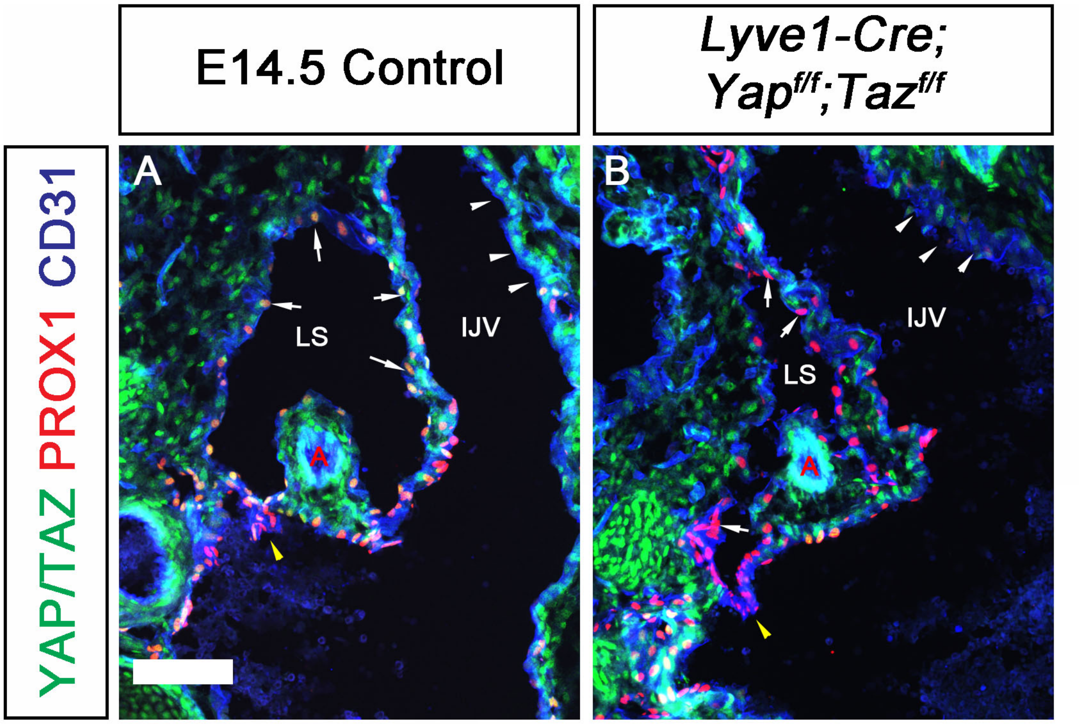

Figure S2: *Lyve1-Cre* efficiently deletes *Yaz/Taz* from LECs.

E14.5 wild type or *Lyve1-Cre;Yap$^{f/f}$;Taz$^{f/f}$* embryos were sectioned and immunostained for the indicated antibodies. Compared to controls expression of YAP/TAZ was reduced in the LECs of mutants (A, B, arrows). YAP/TAZ was only weakly expressed in control LVV-ECs (A, yellow arrowheads) at this stage. LVV-ECs of mutants appeared to be devoid of YAP/TAZ (B, yellow arrowheads).

Measuring bar: (A, B) 100 µm

Statistics: n = 4 for each genotype

Supplementary Figure 3

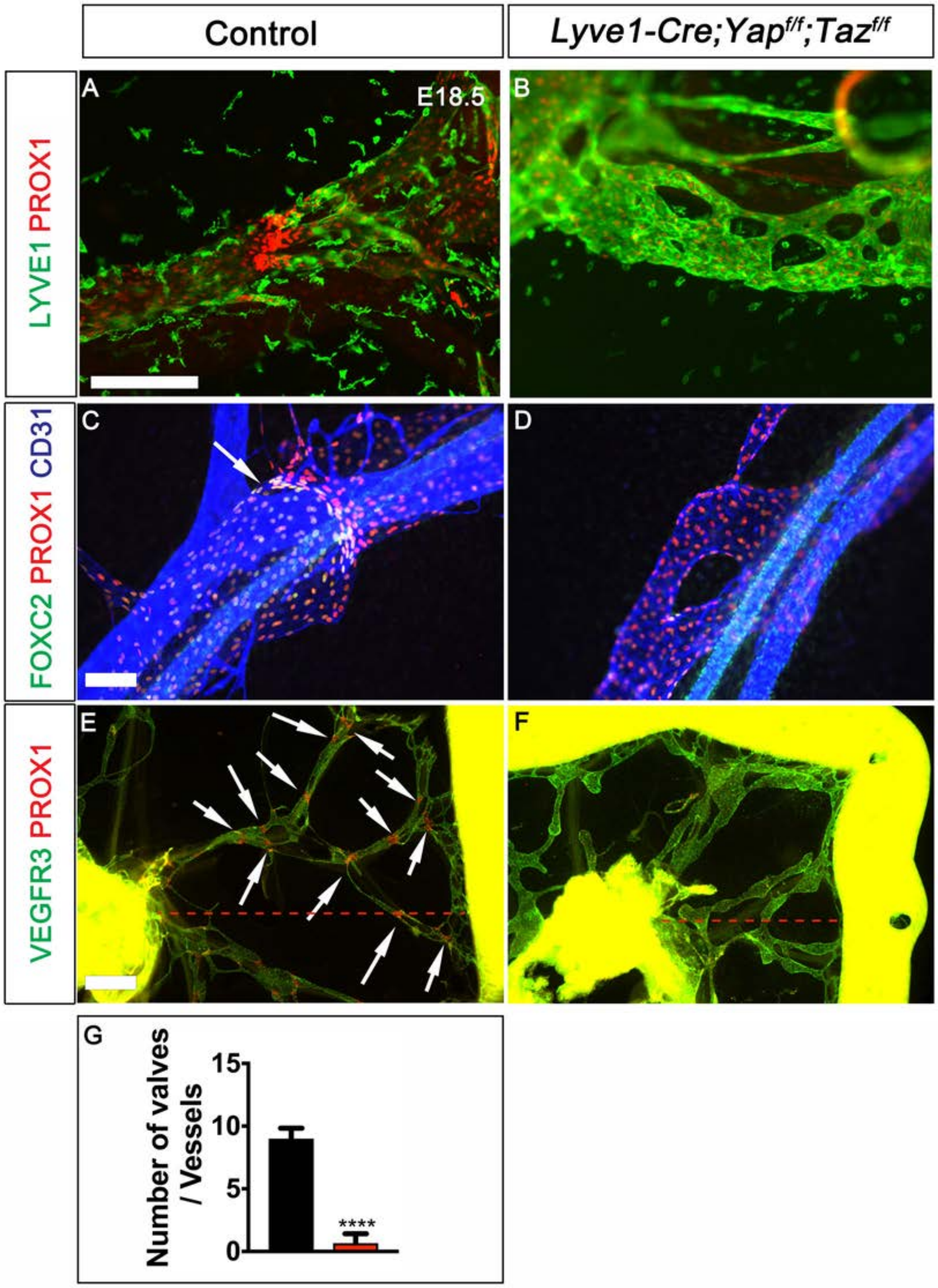

Figure S3: The mesenteric lymphatic vessels of *Lyve1-Cre;Yap$^{f/f}$;Taz$^{f/f}$* embryos are immature and lack LVs.

The guts of E18.5 wild type or *Lyve1-Cre;Yap$^{f/f}$;Taz$^{f/f}$* embryos were analyzed by whole mount IHC using the indicated antibodies. (A-F) The lymphatic vessels of mutants were immature as indicated by the increased expressions of PROX1 (A-D), LYVE1 (A,B) and VEGFR3 (E,F). (C-G) LVs were seen in control embryos (arrows), but not in mutants. Furthermore, the mutant guts were smaller, as indicated by the shorter distance between the central lymph node and the intestinal wall (E, F, red dotted lines).

Measuring bar: (A, B) 200 µm; (C, D) 100 µm; (E, F) 500 µm

Statistics: Statistics: n = 4 for each genotype. **** $P < 0.0001$. Data are presented as mean ± SEM.

Supplementary Figure 4

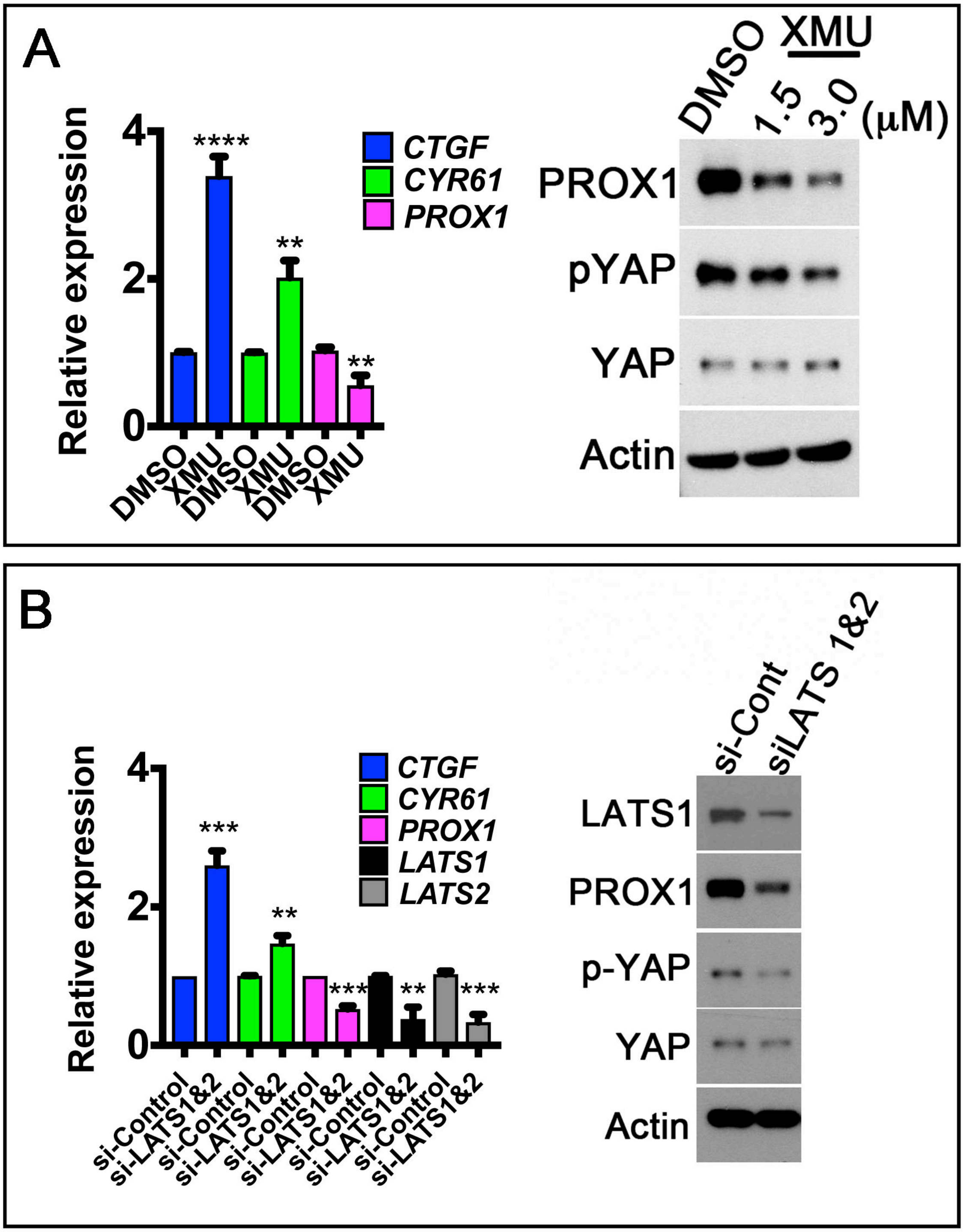

Figure S4: Hyper activation of YAP/TAZ results in the downregulation of PROX1 expression in HLECs.

(A) HLECs were treated with XMU-MP-1, an inhibitor of MST1/2, for 4 hours and the RNA and cell lysate were analyzed for the expression of YAP/TAZ target genes or PROX1 by qRT-PCR or western blot respectively.

(B) HLECs were transfected with siRNA's targeting LATS1/2, grown for an additional 48 hours and their RNA and protein were analyzed as in A.

Statistics: n = 3 for each experiment. ** $P < 0.01$, *** $P < 0.001$, **** $P < 0.0001$. Data are presented as mean ± SEM.

Supplementary Figure 5

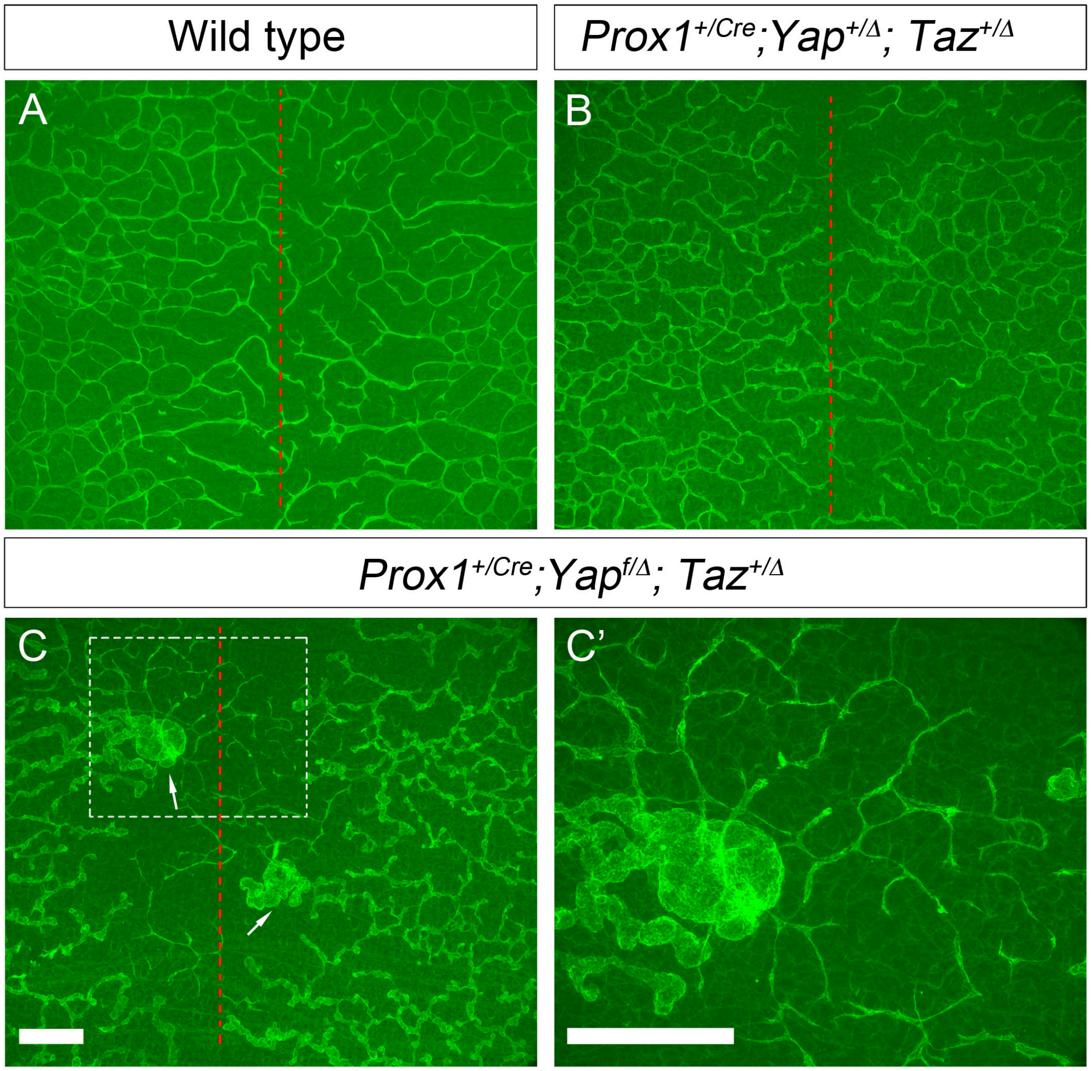

Figure S5: Prox1 genetically interacts with Yap and Taz to regulate lymphatic vascular patterning.

The dorsal skin of E17.5 embryos were analyzed by whole mount IHC for VEGFR3. The red dotted line indicates the midline. The lymphatic vessels of control embryos had crossed the midline (A). Embryos lacking one allele each of *Prox1, Yap* and *Taz* were slightly delayed in their migration (B). In contrast, lymphatic vessels of embryos lacking 3 alleles of *Yap/Taz* in *Prox1*-heterozygous background were severely hypoplastic and had several cystic structures (C, arrows and C').

Statistics: n=3 per genotype.
Measuring bar: 1000 μm.

Development • Supplementary information

Supplementary Figure 6

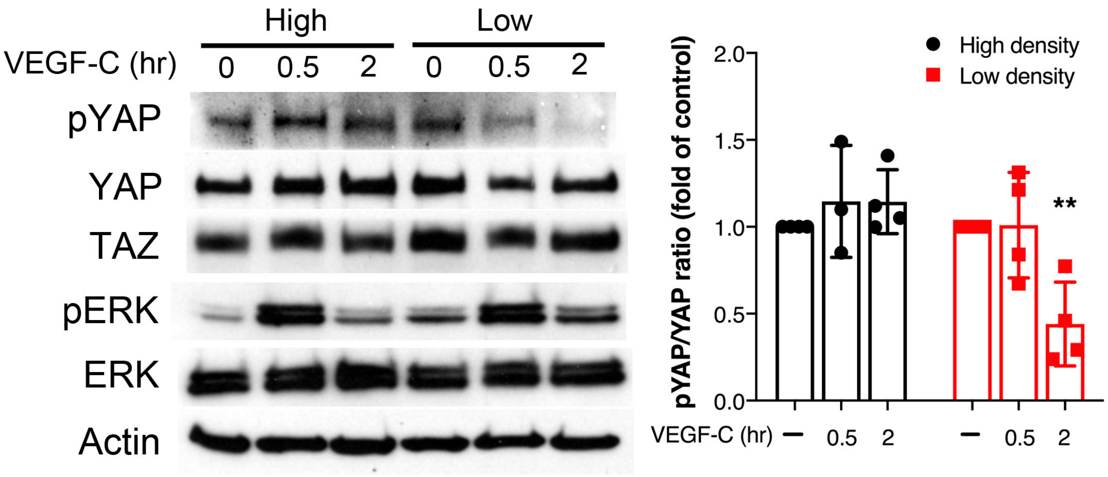

Figure S6: VEGF-C regulates the phosphorylation of YAP in a cell density dependent manner.

HLECs were grown at low cell density (~50% confluency) or high cell density (~100% confluency) and treated with 100 ng/ml VEGF-C for the indicated times. The cell lysate was analyzed by western blot for the indicated antibodies. pYAP/YAP ratio was reduced by VEGF-C in cells grown under low confluency.

Statistics: n=4 per condition. Quantification of western blots is presented as mean ± SD.

Development • Supplementary information

Supplementary Figure 7

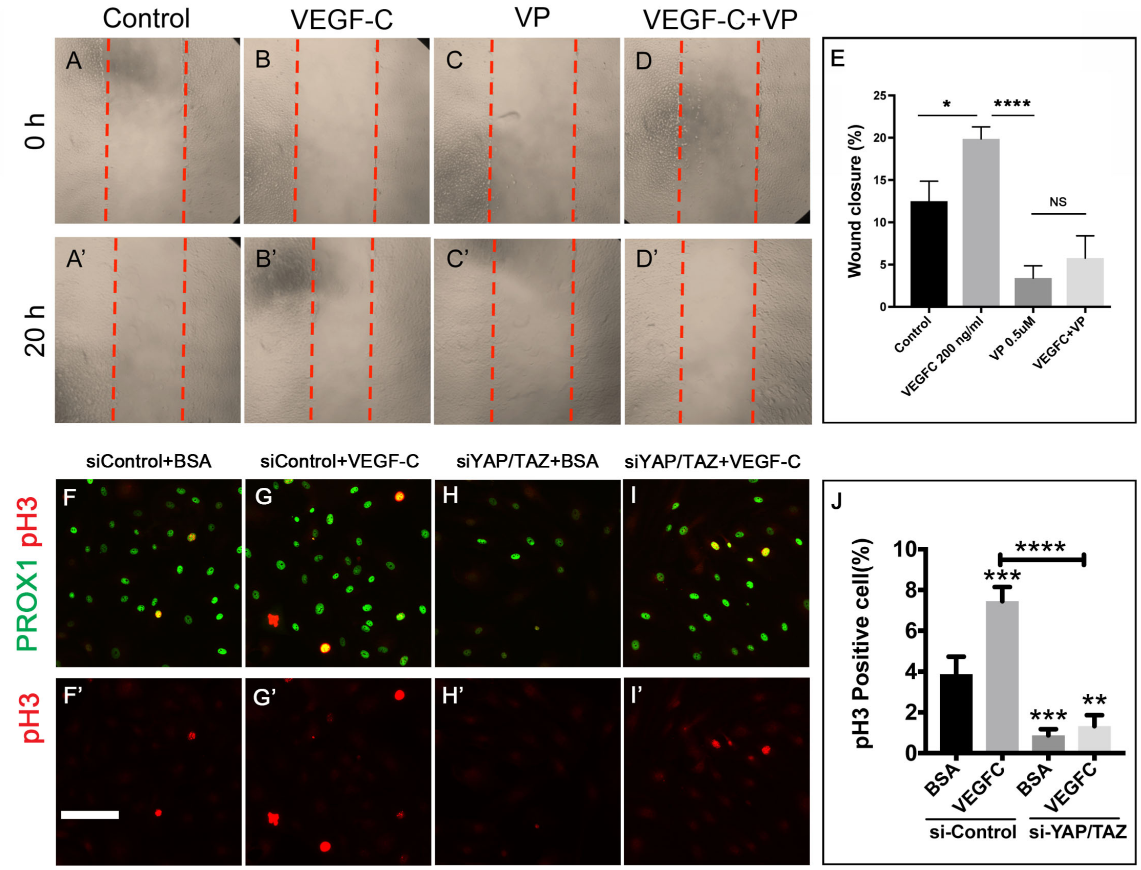

Development • Supplementary information

Figure S7: VEGF-C regulates cell migration and cell proliferation in HLECs in a YAP/TAZ-dependent manner.

(A-E) Wound healing assay was performed using confluent layers of HLECs. The space in between the red dotted lines indicates the cell-free area generated by the scratching. The % reduction in the distance between the red lines after 48 hours was calculated and plotted in E. Compared to untreated cells VEGF-C (100 ng/ml) promoted the migration of HLECs and reduced the size of the wound. In contrast, the YAP/TAZ inhibitor VP inhibited wound healing in the presence or absence of VEGF-C.

(F-J) HLECs transfected with control siRNA or siRNA's targeting YAP and TAZ were cultured in the presence of BSA or VEGF-C (100 ng/ml). The number of proliferating cells was calculated and plotted in J. Knockdown of YAP and TAZ inhibited the proliferation of HLEC's in the presence or absence of VEGF-C.

Statistics: n = 3 for each experiment. * $P < 0.05$, ** $P < 0.01$, *** $P < 0.001$, **** $P < 0.0001$. Data are presented as mean ± SEM.

Measuring bar: 100 μm (F-I).

Supplementary Figure 8

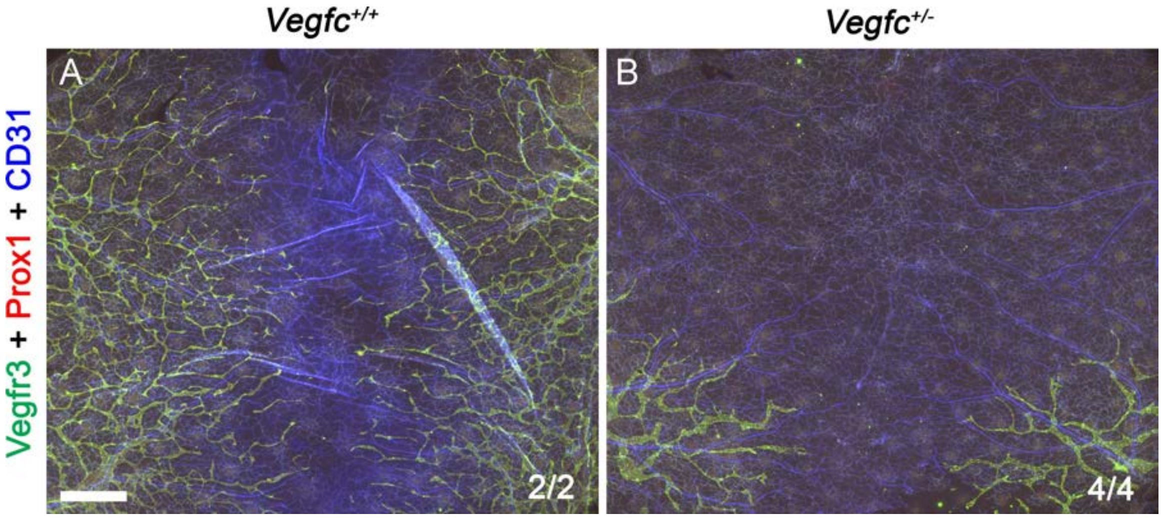

Figure S8: The dermal lymphatic vessels of *Vegfc*$^{+/-}$ mice are hypoplastic.

The dermal vasculature of E15.5 wild type and *Vegfc*$^{+/-}$ embryos were analyzed by immunohistochemistry using the indicated antibodies. (A) The lymphatic vessels of control embryos had migrated from the lateral edges towards the dorsal midline. In contrast, the lymphatic vessels of *Vegfc*$^{+/-}$ embryos were severely hypoplastic and were restricted to the lateral edges.

Measuring bar: 500 µm.

Supplementary Figure 9

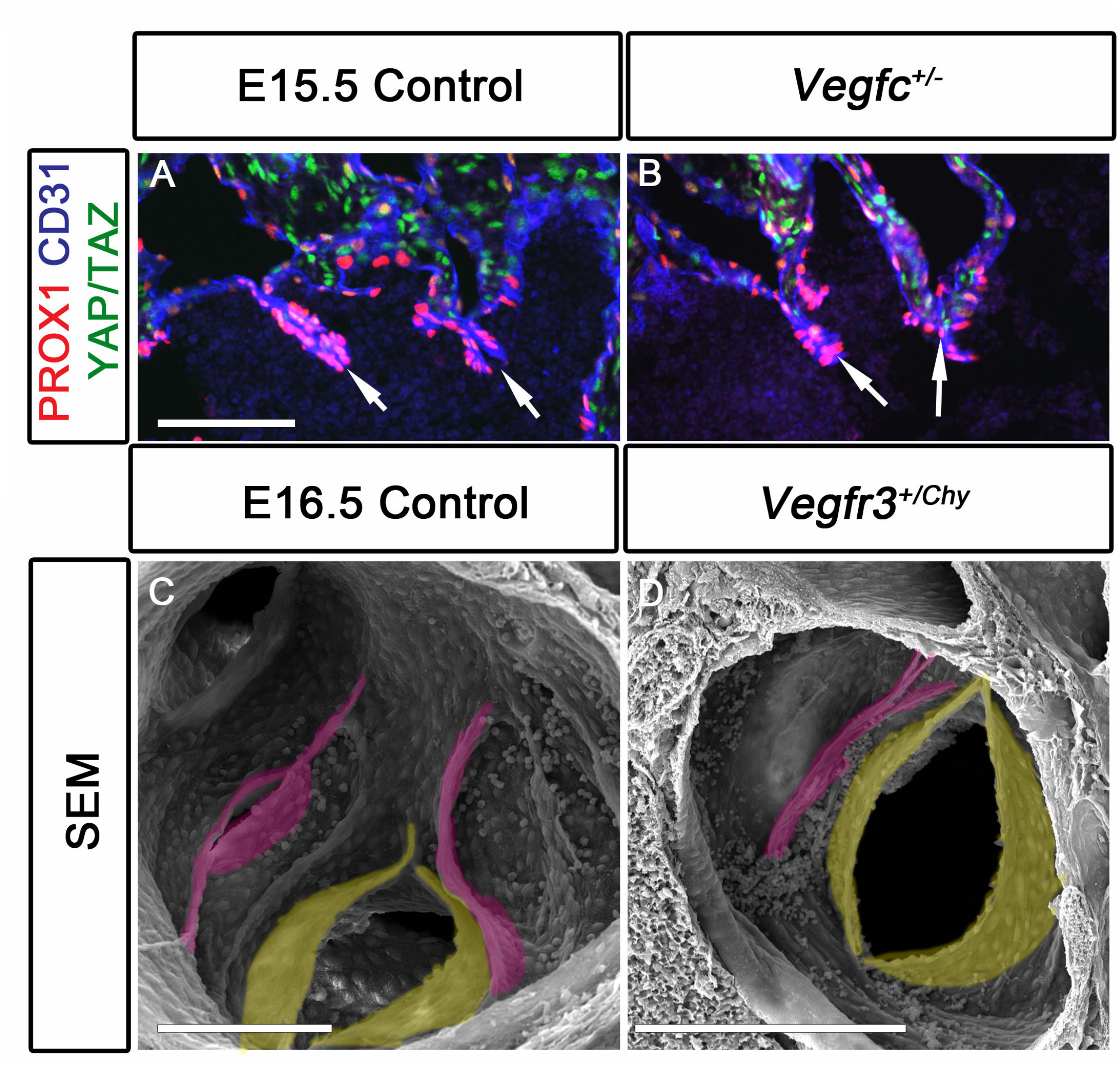

Figure S9: LVVs are observed in *Vegfc*$^{+/-}$ and *Vegfr3*$^{+/chy}$ embryos.

(A, B) LVVs (arrows) were found in both E15.5 wild type and *Vegfc*$^{+/-}$ littermates.

(C, D) LVVs and venous valves (pseudo colored in magenta and yellow respectively) were observed in both E16.5 wild type and *Vegfr3*$^{+/chy}$ littermates.

Statistics: n=4 per genotype.

Measuring bar: (A, B, D) 200 μm; (C) 100 μm.

Development • Supplementary information

Supplementary Figure 10

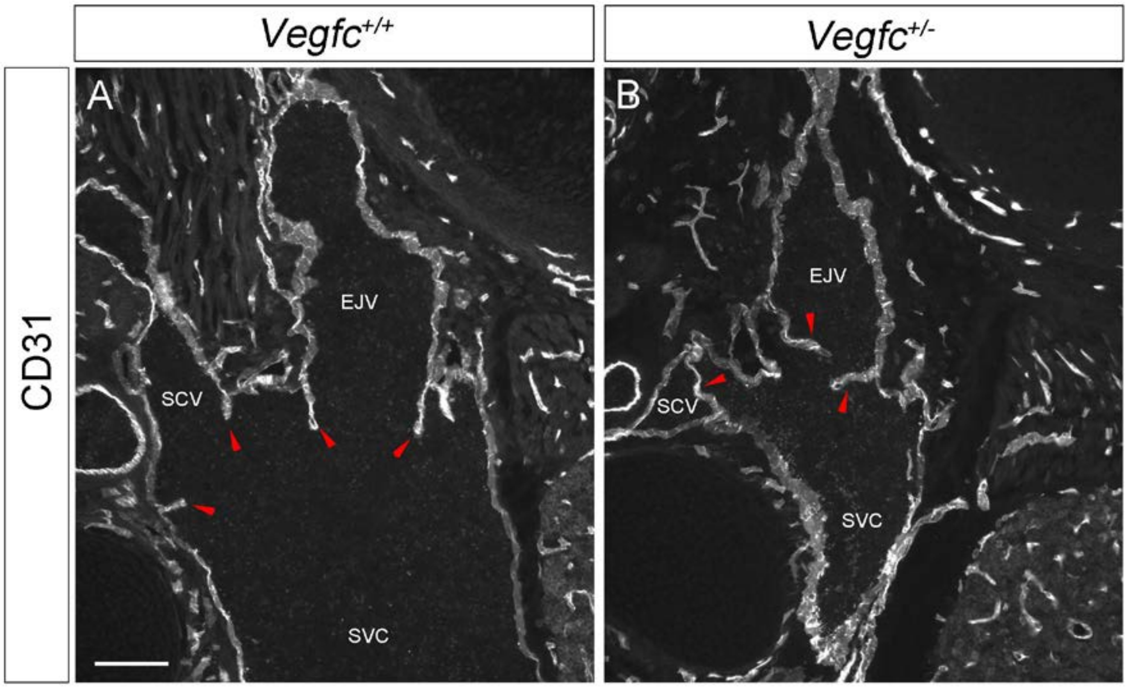

Figure S10: Venous valves are present in *Vegfc*$^{+/-}$ embryos.

(A, B) Venous valves at the junctions of external jugular vein (EJV) and subclavian vein(SCV) with superior vena cava (SVC) were found in both E17.5 wild type and *Vegfc*$^{+/-}$ littermates (arrowheads).

Statistics: n=4 per genotype.

Measuring bar: 200 μm.

Supplementary Figure 11

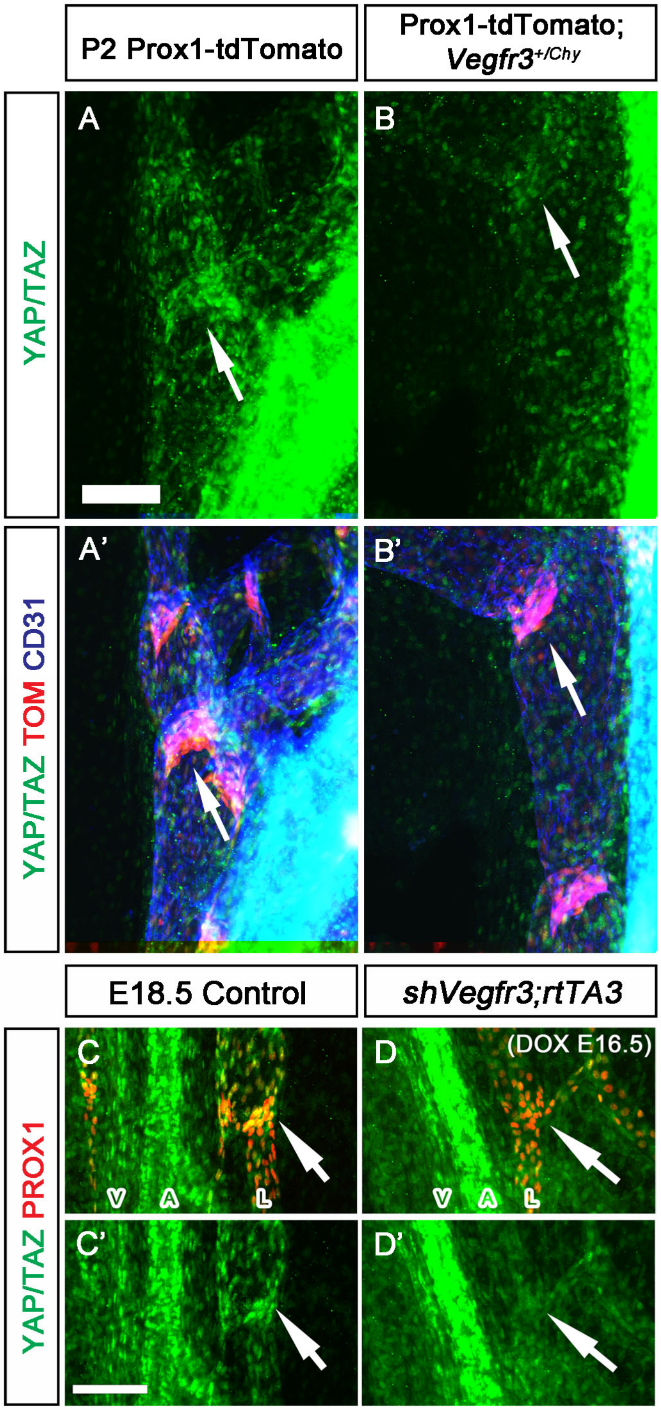

Development • Supplementary information

Figure S11: YAP/TAZ expression is reduced in the LVs of *Vegfr3*$^{+/chy}$ and *shVegfr3;rtTA3* mice.

PROX1^{high} LV rudiments could be observed in the mesenteric lymphatic vessels of P2 *Vegfr3*$^{+/Chy}$ pups or E18.5 *shVegfr3;rtTA3* embryos that were exposed to doxycycline from E16.5 (arrows). However, while YAP/TAZ was observed in the LVs of control embryos, it was downregulated in mutants.

Measuring bar: (A, A', B, B') 100 μm; (C, C', D, D') 200 μm

Statistics: n=4 per genotype.

Table S1. List of differentially expressed genes when DMSO and VP treated HLECs were compared by RNA-seq.

Click here to Download Table S1
