# Supplementary material for: YAP and TAZ maintain PROX1 expression in the developing lymphatic and lymphovenous valves in response to VEGF-C signaling
Source: Development. 2020 Dec 13;147(23):dev195453. doi: 10.1242/dev.195453 (PMC7758626; doi:10.1242/dev.195453)
Supplement: Reviewer comments [file develop-147-195453_review_history.pdf]

YAP and TAZ maintain PROX1 expression in the developing lymphatic and lymphovenous valves in response to VEGF-C signaling

Boksik Cha, YenChun Ho, Xin Geng, Md. Riaj Mahamud, Lijuan Chen, Yeunhee Kim, Dongwon Choi, Tae Hoon Kim, Gwendalyn J. Randolph, Xinwei Cao, Hong Chen and R. Sathish Srinivasan
DOI: 10.1242/dev.195453

Editor: Benoit Bruneau

Review timeline
Original submission: 27 July 2020
Editorial decision: 27 August 2020
First revision received: 8 September 2020
Accepted: 22 September 2020

Original submission

<u>First decision letter</u>

MS ID#: DEVELOP/2020/195453

MS TITLE: YAP and TAZ maintain PROX1 expression in the lymphatic vasculature in response to VEGF-C signaling

AUTHORS: Boksik Cha, YenChun Ho, Xin Geng, Md Riaj Mahamud, Lijuan Chen, Yeunhee Kim, Dongwon Choi, Tae Hoon Kim, Gwendalyn J Randolph, Xinwei Cao, Hong Chen, and R. Sathish Srinivasan

I have now received all the referees' reports on the above manuscript, and have reached a decision. The referees' comments are appended below, or you can access them online: please go to BenchPress and click on the 'Manuscripts with Decisions' queue in the Author Area.

As you will see, the referees express considerable interest in your work, but have some significant criticisms and recommend a substantial revision of your manuscript before we can consider publication. If you are able to revise the manuscript along the lines suggested, which may involve further experiments, I will be happy receive a revised version of the manuscript. Your revised paper will be re-reviewed by one or more of the original referees, and acceptance of your manuscript will depend on your addressing satisfactorily the reviewers' major concerns. Please also note that Development will normally permit only one round of major revision.

We are aware that you may currently be unable to access the lab to undertake experimental revisions. If it would be helpful, we encourage you to contact us to discuss your revision in greater detail. Please send us a point-by-point response indicating where you are able to address concerns raised (either experimentally or by changes to the text) and where you will not be able to do so within the normal timeframe of a revision. We will then provide further guidance. Please also note that we are happy to extend revision timeframes as necessary.

Please attend to all of the reviewers' comments and ensure that you clearly highlight all changes made in the revised manuscript. Please avoid using 'Tracked changes' in Word files as these are lost in PDF conversion. I should be grateful if you would also provide a point-by-point response detailing

© 2020. Published by The Company of Biologists under the terms of the Creative Commons Attribution License (https://creativecommons.org/licenses/by/4.0/).

how you have dealt with the points raised by the reviewers in the 'Response to Reviewers' box. If you do not agree with any of their criticisms or suggestions please explain clearly why this is so.

Reviewer 1

Advance summary and potential significance to field

In this manuscript, Cha et al. explore the lymphatic role of YAP/TAZ signaling in the feedback loop between the master lymphatic regulator, Prox1, and VEGF-C. Utilizing YAP/TAZ deficient mice, the authors provide evidence that YAP/TAZ maintains/promotes Prox1 expression via VEGF-C signaling and that loss of YAP/TAZ leads to the degeneration of LVs and LVVs. Further, their data indicates that VEGF-C signaling is required for lymphatic valve development.

In general, the data presented in this manuscript are of high quality and provide a new outlook on how YAP/TAZ signaling effects PROX1 expression, lymphatic valve development and propagation of the VEGF-C feedback loop to Prox1. Subsequently, these studies implicate a role for YAP/TAZ in maintaining Prox1 expression in lymphatic vessels and valves, contrary to previously published work. In addition, the data presented is fairly compelling in demonstrating a requirement for VEGF-C signaling in lymphatic valve development. However, in my opinion, the manuscript in its current form requires clarifications and additional work to further confirm/substantiate observations, as I have some reservations about certain aspects of the manuscript detailed below.

Comments for the author

MAJOR REMARKS:
1. Some of the main conclusions and results are contradictory to previously published work from Cho et. al, 2018 (Circulation Research). Cho et al. shows that Prox1 expression behaves oppositely to YAP/TAZ (increased YAP/TAZ = reduced Prox1; decreased YAP/TAZ = elevated Prox1), while this manuscript indicates YAP/TAZ and Prox1 go hand in hand during lymphatic vessels development (decreased YAP/TAZ = decreased Prox1). That, in and of itself, is not inherently negative, and may in fact be important to relay to the scientific community. However, these major differences raise several issues within this manuscript that I believe should be addressed.

a. The authors mention that some of their in vitro data showing activated YAP/TAZ signaling reduces Prox1 expression is in agreeance with Cho et al and uses the discussion to provide potential explanations (confluency of cells tested and a zebrafish study). However, aside from these in vitro experiments, the authors completely fail to acknowledge the obvious in vivo discrepancies that exist between the two studies (noted above) and which is a main thrust of the paper (YAP/TAZ regulation of Prox1 expression). At minimum, this should be addressed head on in the discussion section. There could be underlying reasons for the differences, such as different Cre driver lines used and levels of knockdown achieved; the fact that some of the experiments in Cho et al., used Lats1/2 KO mice (although they also confirm their findings in YAP/TAZ mutants); analysis of different vessels/valves (although both assessed dorsal skin vessels). Regardless, the distinctions between the lymphatic studies need to be discussed in detail and properly acknowledged.

b. Given the contrasting results when compared to the Cho et al studies, it is a bit difficult to fully assess the results in this paper, especially since the authors haven’t actually recognized these differences. On one hand, both groups look at the lymphatic vessels in the dorsal skin and observe differences, which are largely confirmed by additional findings in their respective studies. However, different lymphatic vessels or valves are also assessed throughout making it even more difficult to interpret/understand. It would be beneficial if the authors looked at a second similar lymphatic structure, such as the mesenteric lymphatic valves detailed in Cho et al., to assess Prox1 expression compared to controls. Depending on the results of this second comparison, it would help to clarify whether the findings in this paper are more context dependent in the lymphatic vessel/valve assessed or whether the results are ostensibly different. In the latter case, this would also substantially bolster their conclusions and findings that YAP/TAZ maintain/promote Prox1 expression in lymphatic vessels.

© 2020. Published by The Company of Biologists under the terms of the Creative Commons Attribution License (https://creativecommons.org/licenses/by/4.0/).

2. Some of the bioinformatics data used in Figure 4 should be modified for clarification and better representation of the data. For example:
- what are the number of differentially expressed genes in VP treated versus control LECs?
- will the RNA-seq data be made available and where?
- the TEAD4 binding site was "highly conserved", but it is not entirely clear how conserved this site is (the sequence and conservation could be shown) and between what sources are the TEAD4 binding sites considered conserved (human mouse, other cell lines, etc).
- the heat maps in Figure 4C should show multiple samples, not just one sample, since 4 experimental samples were used.

3. In Figure 4, the authors data point to a TEAD4 site approximately 10 kb upstream of the Prox1 gene as being occupied by YAP/TAZ, thereby implicating TEAD4/YAP/TAZ interactions in promoting Prox1 expression. However, there is no additional data indicating whether this site is functionally relevant and regulated by YAP/TAZ to influence Prox1 expression, and if it transcriptionally behaves in a manner consistent with their other findings. Luciferase reporter assays using this H3K27Ac active upstream binding region with a TEAD4 conserved binding site in control and YAP/TAZ siRNA treated LECs would at least further indicate whether this site is transcriptionally regulated by YAP/TAZ and whether it behaves in a manner that is similar to their data or Cho et al.

3. This paper refers to the effects of losing both YAP and TAZ, however, in parts of the paper only one is assessed in a given WB (p-Yap and YAP – Figure 7) or via IHC in vivo (TAZ – Figures 1 and 9). This information could be relevant in terms of relaying its importance and effects on levels of Prox1 expression and because nuances in the differences of expression and/or localization of YAP and TAZ in certain lymphatic vessels may have additional relevancy as noted in Cho et al.

4. If western blots are used to assess the changes in levels of p-YAP/Yap or TAZ expression, which is an important aspect of this paper, multiple experimental samples and quantification of those samples needs to be done. For instance, it appears that Figure 7A,C and D uses western blots from single samples to conclude changes in p-YAP/Yap. As a standard approach this should be done, at least, in triplicate and the protein densities quantified accordingly. On the other hand, Supp Figure 4 indicates an n = 4 was used to perform statistics for the western blots, however no statistical analysis of quantification is presented.

5. In Figure 9, loss of Prox1 in Vegf-C +/- mice doesn't appear to be consistent in the images presented. A-F shows somewhat reduced Prox1 expression levels, while in E-G Prox1 doesn't appear be changed or even possibly up in Vegf-C +/- samples. Representative images should be shown. Along these lines, why is YAP not assessed (see critique 3); levels of YAP/TAZ or p-YAP/YAP are not assessed, yet loss of Vegf-C signaling would be predicted to affect localization and/or levels of p-YAP/YAP. Having this data would further validate the in vitro results in Figure 4.

MINOR REMARKS:

1. The discussion states "Whether YAP/TAZ are required to maintain valves in postnatal mice remains to be determined". Although this is more-or-less accurate, at least to my knowledge, the statement doesn't necessarily capture what is known about YAP/TAZ in lymphatic vessel maintenance. This sentence seems to skirt the results obtained in Cho et al, which showed that YAP/TAZ is dispensable for lymphatic vessel integrity in adults and that activation of YAP/TAZ via Lats1/2 KO leads to dysfunctional lymphatic vessels, including hemorrhagic intestinal lymphatic vessels, blood filled mesenteric lymphatic vessels and defective thoracic duct lymphatic valves.

2. Also in the discussion it states: "However, whether VEGF-C signaling regulates valve development is not known. VEGFR3 is strongly expressed in LVs. Deletion of Vegfc at E14.5 prevented the maturation of collecting lymphatic vessels and the development of LVs within the collecting lymphatics vessel". The first and third sentences appear to be in direct opposition do they not? Perhaps they specifically meant LVV, but this was not clear.

3. Text and figure errors:
- Typo – Figure 2 legend – The scale bars represented in the images do not match the legend (K,L 200μm). I believe it should be (L,M).

© 2020. Published by The Company of Biologists under the terms of the Creative Commons Attribution License (https://creativecommons.org/licenses/by/4.0/).

- Typo – In addition, unlike control embryos, E18.5 Yap/TazLECKO embryos no longer had any LVs (Figure 3H-I) instead of (Figure 3G-I)
- The Figure 3 legend - The scale bars represented in the images do not match the legend (G-H 200μm). I believe it should be (H-I).
- Figure 7B – Label each of the immunofluorescent image panels with the specific conditions (VEGF-C - and VEGF-C +)
- Figure 7D – In the western blot data. Indicate VEGF-C beside the time points to show what factor was used during that specific stimulation study
- Figure 9 E – there is expression of Prox1 in the merged image, however in the image E''- there is no PROX1 expression?

Reviewer 2

Advance summary and potential significance to field

The lymphatic system is a key component of the circulation system and plays various important functions in animals. Malformation of lymphatic vasculature often leads to lymphedema. Unlike development of many other organs/systems, the mechanisms that regulate lymphatic vascular development remain largely unclear. In the manuscript by Cha et al., the authors report a novel regulatory loop that controls the development of lymphatic valves, lymphovenous valves, and venous valves during mouse embryonic development.

Before this work, it has been reported that PROX1, the master regulator of lymphatic system, and the VEGF-C/VEGFR3 signaling pathway form a regulatory loop. This regulatory loop acts in the lymphatic endothelial cells to regulate lymaphtic vascular development. At the molecular level, how this regulatory loop operates remain unknown. Results presented here argue that YAP and TAZ are critical mediators that act downstream of VEGF-C signaling to regulate the expression of PROX1 expression during lymphatic vascular development.

The conclusion of the paper is convincing, supported by high quality results. The manuscript is carefully drafted. In my opinion, this work represents an important contribution the field.

Comments for the author

Before this work, it has been reported that PROX1, the master regulator of lymphatic system, and the VEGF-C/VEGFR3 signaling pathway form a regulatory loop. This regulatory loop acts in the lymphatic endothelial cells to regulate lymaphtic vascular development. At the molecular level, how this regulatory loop operates remain unknown. Results presented here argue that YAP and TAZ are critical mediators of the PROX1 expression in response to VEGF-C signaling during lymphatic vascular development. The authors started from analyzing the expression/activity of YAP and TAZ during valve maturation. Results show that the activity of YAP and TAZ gradually increases in developing LVs and LVVs. Motivated by this finding, the authors used genetic approach to conditionally and constitutively delete YAP/TAZ in the LEC progenitors. By analyzing the morphology of the mutant embryos, performing IF for markers, and SEM, they found YAP and TAZ are not required for the initiation of lymphovenous valves, but play an important role in their maintenance. To figure out the molecular mechanism by which YAP and TAZ regulate valve development, they carried out analyses in Human Lymphatic Endothelial Cells (HLECs). RNAseq analysis shows that several pathways critical for vascular development were affected by pharmaceutical inhibition of YAP/TAZ. Regulation of Prox1 YAP/TAZ was further validated by siRNA-based knockdown experiment. Furthermore, the authors identified a TEAD4 binding sequence from the regulatory element of the prox1 gene and validated it by ChIP assay. To determine if YAP/TAZ regulate Prox1 in vivo during mouse lymphatic vascular development, they assessed Prox1 expression in the LECs and LVV-ECs. Indeed, downregulation of Prox1 was detected in mutant embryos deficient in YAP/TAZ. Moreover, deletion of YAP and TAZ aggravates the lymphatic vascular defects associated with Prox1-heterozgosity. The authors further studied the link between YAP/TAZ and VEGF-C signaling during lymphatic vascular development. Results show that in both HLECs and HEK293T cells, VEGF-C induces phosphorylation and nuclear localization of YAP, and the expression of PROX1 at both RNA and protein levels. By analyzing VEGF-C mutants, the expression of YAP/TAZ targets is downregulated in the LVs of Vegfc+/- embryos and VEGF-C regulates the

© 2020. Published by The Company of Biologists under the terms of the Creative Commons Attribution License (https://creativecommons.org/licenses/by/4.0/).

formation of LVVs. Based on these findings, the authors conclude that YAP and TAZ maintain PROX1 expression in the lymphatic vasculature in response to VEGF-C signaling.

Overall, the key finding of this work is novel. Conclusion of the paper is supported by solid experimental evidence. The manuscript is organized and clearly written. I very much like this work. I have only one minor point. I would suggest the authors to quantify the western blot in Figure 7A and present a bar graph.

Reviewer 3

Advance summary and potential significance to field

The paper by Cha et al investigates the role of YAP and TAZ in lymphatic development using mice. They initially utilise a Lyve1-Cre mediated model of YAP/TAZ knockout that deletes YAP/TAZ from approximately E10.5 onwards and show that YAP/TAZ are essential for vessel morphogenesis during dermal lymphangiogenesis and the formation of LVVs. They show that in the absence of YAP/TAZ, lymphatic vessels have lower levels of PROX1 than normal in developing embryos. In vitro, they go on to show that either using pharmacological or knockdown approaches in LECs, loss of YAP/TAZ function leads to reduced expression of PROX1 and a number of other vascular markers. They also showed that TEAD4 (DNA binding Yap-cofactor) binds upstream of the PROX1 transcription start site and that activation of YAP/TAZ leads to increased PROX1 and YAP/TAZ Target genes.
Genetic interaction data is also presented and suggested to indicate genetic interactions between YAP/TAZ, PROX1 and VEGFC/VEGFR3 at the level of different lymphatic phenotypes. In vitro epistasis data suggests that VEGFC increases PROX1 levels in a YAP/TAZ dependent manner and that VEGFC driven proliferation is YAP/TAZ dependent. Importantly, they show that VEGFC+/- heterozygous animals show reduced Prox1 levels and reduced YAP/TAZ target genes. This suggests that YAP/TAZ are essential downstream of VEGFC including in the activation of PROX1.

Overall, the work utilises an impressive array of genetic models and tools. It is quite important as it is proposing a very different model to that recently published by the Koh laboratory (Cho et al 2019). In fact, the previous work suggests that YAP/TAZ repress PROX1, while this study suggests they promote PROX1 expression Â– quite different findings indeed. On this point, it is important that the authors take the time to highlight the technical differences between the two studies and provide a reasonable explanation for the differences (see below).
Overall, the data here are generally quite convincing and so this may significantly clarify our understanding of how Hippo signalling controls lymphatic development.

Comments for the author

The paper covers a lot of territory and with the many experimental approaches used, there are several technical concerns throughout that the authors should address. This reviewer is also concerned about the accuracy of a few of the major conclusions that are being drawn and whether they are fully supported by the data (see below). In general, the work could benefit from more explanation of experimental detail throughout. Nevertheless, it is overall an interesting and important study that will change our view of how the Hippo pathway and YAP/TAZ function in lymphatic development if a number of issues are addressed.

Concerns to be addressed:

1. The data in Figure 3 is strikingly different to the data shown in the previous paper by Cho et al 2019. In that study, the authors knocked out YAP and TAZ with Prox1-CreErt2 and found a strong up-regulation of PROX1 in large dilated and dysmorphic dermal lymphatics by E15.5. Here the authors see relatively minor defects by E16.5 and potentially a decrease in PROX1 in dermal lymphatics. As the only difference seems to be the driver Cre strain, could the difference be due to inefficient deletion in the Lyve1-Cre strain? Have the authors validated directly that their Lyve1-Cre deletor is efficiently knocking out both YAP and TAZ? If so they need to show the data.
In this context, it is interesting to note that when the authors did use a Prox1-Cre strain in Figure 6 and Supp 3 combined with their KO line, they did start to see the same sort of structural defects reported by Cho et al 2019.

© 2020. Published by The Company of Biologists under the terms of the Creative Commons Attribution License (https://creativecommons.org/licenses/by/4.0/).

Finally, as well as the technical issue above, the paper would be further improved if the authors provided more discussion on the experimental differences that could explain the different conclusions in the two papers.

2. The increase of Endomucin in the lymph sacs of Prox1-Cre YAP/TAZ KO mice may be indicative of a change in LEC fate to a blood vascular like fate. However, this is a big claim to be based on just one marker. Can the authors confirm this with additional markers? Is this phenotype present earlier than E14.5 and if so doesn't this suggest that in fact YAP/TAZ do play a role in specification and LEC fate but that the late deletion using Lyve1-Cre is the reason for no obvious early specification defects? A more clear discussion of the timing of knockout relative to key events in mouse lymphatic development is needed.

3. The authors conclude that "our data suggest that YAP and TAZ are not required for the initial specification of LECs" however they delete from E10.5 well after the initial specification of LECs at the level of the CV. They would need to delete before specification to test the hypothesis that YAP/TAZ influence specification. As such they should revise their conclusion about specification or perform additional analyses (as point 2).

4. In Figure 3 H vs I Prox1 expression looks to be at normal levels in panel I except for the failure of the lymphatics to form valve territories (which express high PROX1). If fluorescence intensity is quantified in the KO LEC nuclei vs non valve territory LECs, is there a change in expression levels that fits with the in vitro suggestion of YAP-TAZ regulating PROX1? (obviously any quantification would need to be normalised and control for variation in staining from embryo to embryo)

5. The claim that "the transcriptional co-activators YAP and TAZ are required to maintain PROX1 expression in response to VEGF-C signalling" seems to be an overstatement. This also impacts the title of the paper. The authors conclude that "Together these results suggest that YAP/TAZ are necessary to maintain PROX1 expression in developing lymphatic vasculature" in relation to the analysis of Prox1-tdTomato levels in Figure 5. The reduction in Prox1 levels is really quite subtle – the graph in figure 5 shows a reduction by about ~30% based on q-PCR and this is less of a reduction than for YAP/TAZ targets. Based on that, a fair conclusion looking at this data would be that Prox1 is still maintained in the absence of YAP/TAZ at both the protein and the transcriptional level but that VEGFC enhances PROX1 expression in a YAP/TAZ dependent manner.

6. How specific is the response to the drug VP in cultured LECs? Are non-Hippo targets unchanged? How many genes overall were changed in the RNA seq analysis and were pathways associated with cell death or drug toxicity changed? A full bioinformatic analysis and QC should be provided in supp material with the RNA seq data to give the reader confidence that the analysis was clean and the observations presented are specific.

7. For immuno-fluorescence stains such as in Supp Figure 1; it is very difficult to appreciate if the staining for YAP/TAZ or CTGF is specific. Can the authors show a wider field of view for the stains to indicate if there is tissue specificity? How were these controlled for specificity?

8. Figure 8 seems to lack careful quantification of the phenotypes, making it hard to interpret how representative the images provided are. N- values for the number of embryos examined are given, but how many LVV forming cells were scored per embryo of each genotype is unclear. Likewise, for dermal lymphatics and venous valve cells. Further quantification is needed to strengthen the data.

9. In Figure 1, the Taz staining looks pan-endothelial. Is this the case? Or is there quantifiably some restriction or enrichment in the valve domains? Please show evidence of restriction to valves if that is the claim.

10. Is Lyve1 really exclusively a LEC marker in ECs? If not, then the double KO model should not be referred to as LECKO and expression in other vessels, such as veins, should be noted clearly as a caveat to these analyses.

11. There is no legend for Figure 2I and so it is unclear what was scored here (number of ECs per valve? Number of valves per embryo?) and what the graph represents.

© 2020. Published by The Company of Biologists under the terms of the Creative Commons Attribution License (https://creativecommons.org/licenses/by/4.0/).

12. Statistics – the replicates are given at the end of each figure legend but it is unclear if the replicates are technical or biological, particularly for in vitro data. Please clarify in detail in the methods.

First revision

Author response to reviewers' comments

We thank the reviewers for their encouraging comments and confidence in our results. We have attempted to address all their questions and concerns in the revised manuscript. Our point-by-point responses to their comments are provided below.

Based on the discussions below we have changed the title of the manuscript to **YAP and TAZ maintain PROX1 expression in the developing lymphatic and lymphovenous valves in response to VEGF-C signaling.**

Reviewer 1
In this manuscript, Cha et al. explore the lymphatic role of YAP/TAZ signaling in the feedback loop between the master lymphatic regulator, Prox1, and VEGF-C. Utilizing YAP/TAZ deficient mice, the authors provide evidence that YAP/TAZ maintains/promotes Prox1 expression via VEGF-C signaling and that loss of YAP/TAZ leads to the degeneration of LVs and LVVs. Further, their data indicates that VEGF-C signaling is required for lymphatic valve development.

In general, the data presented in this manuscript are of high quality and provide a new outlook on how YAP/TAZ signaling effects PROX1 expression, lymphatic valve development and propagation of the VEGF-C feedback loop to Prox1. Subsequently, these studies implicate a role for YAP/TAZ in maintaining Prox1 expression in lymphatic vessels and valves, contrary to previously published work. In addition, the data presented is fairly compelling in demonstrating a requirement for VEGF-C signaling in lymphatic valve development. However, in my opinion, the manuscript in its current form requires clarifications and additional work to further confirm/substantiate observations, as I have some reservations about certain aspects of the manuscript detailed below.

Reviewer 1 Comments for the Author:

MAJOR REMARKS:
1. Some of the main conclusions and results are contradictory to previously published work from Cho et. al, 2018 (Circulation Research). Cho et al. shows that Prox1 expression behaves oppositely to YAP/TAZ (increased YAP/TAZ = reduced Prox1; decreased YAP/TAZ = elevated Prox1), while this manuscript indicates YAP/TAZ and Prox1 go hand in hand during lymphatic vessels development (decreased YAP/TAZ = decreased Prox1). That, in and of itself, is not inherently negative, and may in fact be important to relay to the scientific community. However, these major differences raise several issues within this manuscript that I believe should be addressed.

a. The authors mention that some of their in vitro data showing activated YAP/TAZ signaling reduces Prox1 expression is in agreeance with Cho et al and uses the discussion to provide potential explanations (confluency of cells tested and a zebrafish study). However, aside from these in vitro experiments, the authors completely fail to acknowledge the obvious in vivo discrepancies that exist between the two studies (noted above) and which is a main thrust of the paper (YAP/TAZ regulation of Prox1 expression). At minimum, this should be addressed head on in the discussion section. There could be underlying reasons for the differences, such as different Cre driver lines used and levels of knockdown achieved; the fact that some of the experiments in Cho et al., used Lats1/2 KO mice (although they also confirm their findings in YAP/TAZ mutants); analysis of different vessels/valves (although both assessed dorsal skin vessels). Regardless, the distinctions between the lymphatic studies need to be discussed in detail and properly acknowledged.

© 2020. Published by The Company of Biologists under the terms of the Creative Commons Attribution License (https://creativecommons.org/licenses/by/4.0/).

Thank you for your suggestion. We have now added the following to the *Discussion*.

>>>> In contrast to our findings Cho et al showed that YAP and TAZ inhibit PROX1 expression in the developing lymphatic vessels (Cho et al., 2019). The differences could be due to the distinct Cre lines that were used. While we used *Lyve1-Cre*, Cho et al used Prox1-CreERT2 (Bazigou et al., 2011). While Cho et al observed severe edema in their mutant embryos we did not observe edema in most of our samples. The more severe phenotype observed by Cho et al could be due to more potent/rapid gene deletion by Prox1-CreERT2. As mentioned above we suspect that YAP/TAZ play a primary role in maintaining the integrity of LVs and LVVs. Degeneration of LVVs at an earlier stage in the *Yap/Taz*^{LECKO} embryos generated by Cho et al might have affected lymphatic drainage earlier, which in turn would have prevented lymphatic vessel maturation resulting in sustained expression of PROX1. Deleting *Yap/Taz* at various developmental time points and in specific compartments of the lymphatic vasculature (LEC progenitors, LECs, LVs, LVVs, tip cells, stalk cells) could test these possibilities and provide better resolution of YAP/TAZ activity.

There are also differences in in vitro data generated by Cho et al and us. While, we have determined that inhibition of YAP/TAZ activity by VP or siRNA inhibits PROX1 expression, results generated by Cho et al make the opposite conclusion. It is possible that these differences are due to differences in cell lines or culture conditions that were used. Nevertheless, there are important points of congruity between our findings and Cho et al. Specifically, we also found that over-activation of YAP/TAZ, via pharmacological inhibition of MST1/2 or RNAi-mediated depletion of LATS1/2 reduces PROX1 expression in confluent HLECs. These results—both loss and over-activation of YAP/TAZ could result in the down regulation of PROX1 expression in the lymphatic vasculature—suggest that a precise level of YAP/TAZ activity regulates PROX1 expression.

b. Given the contrasting results when compared to the Cho et al studies, it is a bit difficult to fully assess the results in this paper, especially since the authors haven't actually recognized these differences. On one hand, both groups look at the lymphatic vessels in the dorsal skin and observe differences, which are largely confirmed by additional findings in their respective studies. However, different lymphatic vessels or valves are also assessed throughout making it even more difficult to interpret/understand. It would be beneficial if the authors looked at a second similar lymphatic structure, such as the mesenteric lymphatic valves detailed in Cho et al., to assess Prox1 expression compared to controls. Depending on the results of this second comparison, it would help to clarify whether the findings in this paper are more context dependent in the lymphatic vessel/valve assessed or whether the results are ostensibly different. In the latter case, this would also substantially bolster their conclusions and findings that YAP/TAZ maintain/promote Prox1 expression in lymphatic vessels.

We have quantified the fluorescent intensities of Prox1-tdTomato and PROX1 in the dermal lymphatic vessels. As shown in the **Figure 6G**, the most striking defect in *Lyve1-Cre;Yap*$^{f/f}$*;Taz*$^{f/f}$ embryos is the absence of PROX1^{high} LVs. While tdTomato expression is indeed downregulated PROX1 expression does not appear to be obviously changed. This data strongly indicates that the primary role of YAP/TAZ is in maintaining PROX1 expression within LVs and LVVs.

We have also analyzed the mesenteric lymphatics as suggested. The mesenteric lymphatic vessels of LVs. We have now added this data as **Supplementary Figure 3**. As shown here in **Figure 1 for reviewers** PROX1 expression is indeed upregulated in the mutant LECs. However, we are hesitant to make strong conclusions based on this data as we and others *Lyve1-Cre;Yap*$^{f/f}$*;Taz*$^{f/f}$ embryos are immature and lack have shown in the past that *Lyve1-Cre* is expressed in the blood vascular endothelial cells of the gut (Geng et al., 2020, Dellinger et al., 2015). In fact, the gut of mutant embryos is smaller in size compared to controls. Hence, defects in the mesenteric lymphatic vessels could be secondary to blood vascular defects.

Nevertheless, our data clearly indicates that LVs and LVVs degenerate in the absence of YAP/TAZ. Furthermore, YAP/TAZ expression and activity (as indicated by CTGF expression) are enriched in the valves. Therefore, we have revised our manuscript to focus on YAP/TAZ activity in the valves. We suggest that YAP/TAZ regulate the integrity of valves by maintaining PROX1 expression. Any change in PROX1 expression in the lymphatic vessels could be due to defects in lymphatic drainage caused by the degeneration of valves.

© 2020. Published by The Company of Biologists under the terms of the Creative Commons Attribution License (https://creativecommons.org/licenses/by/4.0/).

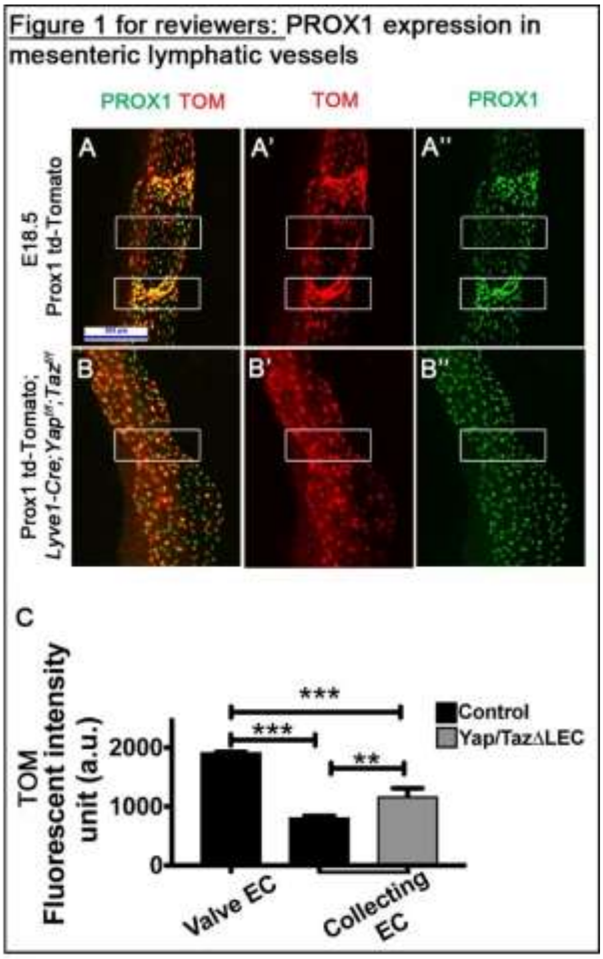

Figure 1 for reviewers: PROX1 expression in mesenteric lymphatic vessels

2. Some of the bioinformatics data used in Figure 4 should be modified for clarification and better representation of the data. For example:
- what are the number of differentially expressed genes in VP treated versus control LECs?
- will the RNA-seq data be made available and where?
- the TEAD4 binding site was "highly conserved", but it is not entirely clear how conserved this site is (the sequence and conservation could be shown) and between what sources are the TEAD4 binding sites considered conserved (human, mouse, other cell lines, etc).
- the heat maps in Figure 4C should show multiple samples, not just one sample, since 4 experimental samples were used.

1. We are now providing the entire RNA-seq data as **Supplementary Table 1**. In this file we have also separated the genes based on the significance of differential expression, which are further classified as upregulated or downregulated genes with Log fold change>1.

2. We have revised the heat map as suggested.

3. We have provided a picture of conservation of TEAD4 binding site across various species.

3. In Figure 4, the authors data point to a TEAD4 site approximately 10 kb upstream of the Prox1 gene as being occupied by YAP/TAZ, thereby implicating TEAD4/YAP/TAZ interactions in promoting Prox1 expression. However, there is no additional data indicating whether this site is functionally relevant and regulated by YAP/TAZ to influence Prox1 expression, and if it transcriptionally behaves in a manner consistent with their other findings. Luciferase reporter assays using this H3K27Ac active upstream binding region with a TEAD4 conserved binding site in control and YAP/TAZ siRNA treated LECs would at least further indicate whether this site is transcriptionally regulated by YAP/TAZ and whether it behaves in a manner that is similar to their data or Cho et al.

© 2020. Published by The Company of Biologists under the terms of the Creative Commons Attribution License (https://creativecommons.org/licenses/by/4.0/).

This is a valid, yet difficult question to address. As the reviewer most likely knows luciferase report assays for enhancer analysis are highly artificial. Reporter gene activation depends on numerous conditions such as the promoter elements used in the plasmids, cell lines, transfection efficiency and culture conditions. Importantly, transcription factors work in synergy with each other and we simply do not have a full understanding of the various transcription factors that are required for YAP/TAZ activity. As we show in Figure 5C and E, there is a conserved GATA2 binding site in the vicinity of TEAD4 binding site. Also present in this region are FOS, JUN and TCF7L2 sites. The presence or absence of these transcription factors could significantly impact the activity of YAP/TAZ. Furthermore, transcription factors tend to associate with more than one site in the regulatory element of their target genes, and there tends to be compensation for the loss of one regulatory element from others. Taking into consideration all these potential limitations we do not think that we will be able to perform this assay and generate an interpretable result in a reasonable amount of time. An unbiased ChIP-seq screen in HLECs followed by CRISPR/Cas9 mediated deletion in HLECs or in mice is required to decisively demonstrate the importance of regulatory elements. As the reviewer will surely appreciate this will be a major project on it's own.

Nevertheless, considering the limitation of our work we have rephrased our conclusion in the manuscript. I hope the reviewer finds this modification reasonable and acceptable.

>>>We identified a conserved TEAD4 binding site (**Figure 5D**) within the regulatory elements upstream of *PROX1* (**Figure 5E**), and ChIP-PCR revealed that YAP binds to this site in HLECs (**Figure 5F**). Together these data suggest that YAP/TAZ cooperates with TEAD4 to directly activate PROX1 expression in HLECs. However, the functional significance of this binding site is currently unknown, and it is likely that YAP/TAZ associates with multiple sites in the distal regulatory elements of genes as described previously (Galli et al., 2015 and Stein et al., 2015).

4. This paper refers to the effects of losing both YAP and TAZ, however, in parts of the paper only one is assessed in a given WB (p-Yap and YAP – Figure 7) or via IHC in vivo (TAZ – Figures 1 and 9). This information could be relevant in terms of relaying its importance and effects on levels of Prox1 expression and because nuances in the differences of expression and/or localization of YAP and TAZ in certain lymphatic vessels may have additional relevancy as noted in Cho et al.

We have now added TAZ western blot data to the new **Figure 8** and YAP/TAZ IHC data to the new **Supplementary Figure 9** (corresponding to new **Figure 9**).

5. If western blots are used to assess the changes in levels of p-YAP/Yap or TAZ expression, which is an important aspect of this paper, multiple experimental samples and quantification of those samples needs to be done. For instance, it appears that Figure 7A,C and D uses western blots from single samples to conclude changes in p-YAP/Yap. As a standard approach, this should be done, at least, in triplicate and the protein densities quantified accordingly. On the other hand, Supp Figure 4 indicates an n = 4 was used to perform statistics for the western blots, however no statistical analysis of quantification is presented.

We have now quantified the western blots in **Figure 8** and **Supplementary Figure 6**.

6. In Figure 9, loss of Prox1 in Vegf-C +/- mice doesn't appear to be consistent in the images presented. A-F shows somewhat reduced Prox1 expression levels, while in E-G Prox1 doesn't appear be changed or even possibly up in Vegf-C +/- samples. Representative images should be shown. Along these lines, why is YAP not assessed (see critique 3); levels of YAP/TAZ or p-YAP/YAP are not assessed, yet loss of Vegf-C signaling would be predicted to affect localization and/or levels of p-YAP/YAP. Having this data would further validate the in vitro results in Figure 4.

1. We have now added a **Supplementary Figure 9** in which we have performed IHC for YAP/TAZ in Chy and shFLT4 mice. I hope this data is acceptable. The Vegfc+/- mice are difficult to generate due to their severe lymphatic defects and many of them were lost during the pandemic related shut down.

2. We mistakenly failed to include the PROX1 layer while assembling old Fig 9E''. We have now corrected it. We want to point out that qPCR for Prox1 and other Yap/Taz target genes was

© 2020. Published by The Company of Biologists under the terms of the Creative Commons Attribution License (https://creativecommons.org/licenses/by/4.0/).

performed using LECs sorted from Vegfc+/- embryos (old Figure 9H, new Figure 10H) to address this possibility definitively.

MINOR REMARKS:

1. The discussion states "Whether YAP/TAZ are required to maintain valves in postnatal mice remains to be determined". Although this is more-or-less accurate, at least to my knowledge, the statement doesn't necessarily capture what is known about YAP/TAZ in lymphatic vessel maintenance. This sentence seems to skirt the results obtained in Cho et al, which showed that YAP/TAZ is dispensable for lymphatic vessel integrity in adults and that activation of YAP/TAZ via Lats1/2 KO leads to dysfunctional lymphatic vessels, including hemorrhagic intestinal lymphatic vessels, blood filled mesenteric lymphatic vessels and defective thoracic duct lymphatic valves.

Thank you for your comment. We have now removed the statement.

2. Also in the discussion it states: "However, whether VEGF-C signaling regulates valve development is not known. VEGFR3 is strongly expressed in LVs. Deletion of Vegfc at E14.5 prevented the maturation of collecting lymphatic vessels and the development of LVs within the collecting lymphatics vessel". The first and third sentences appear to be in direct opposition do they not? Perhaps they specifically meant LVV, but this was not clear.

Thank you for your suggestion. We have now edited this statement as following.

VEGFR3 is strongly expressed in LVs (Norrmen et al., 2009). Deletion of *Vegfc* at E14.5 prevented the maturation of collecting lymphatic vessels and the development of LVs within the collecting lymphatic vessels (Nurmi et al., 2015). However, as the lymphatic vessels of these mice were thinner and immature, whether VEGF-C directly or indirectly regulates LV development remains unknown.

3. Text and figure errors:

-Typo – Figure 2 legend – The scale bars represented in the images do not match the legend (K,L 200µm). I believe it should be (L,M).
-Typo – In addition, unlike control embryos, E18.5 Yap/TazLECKO embryos no longer had any LVs (Figure 3H-I) instead of (Figure 3G-I)
-The Figure 3 legend - The scale bars represented in the images do not match the legend (G-H 200µm). I believe it should be (H-I).
-Figure 7B – Label each of the immunofluorescent image panels with the specific conditions (VEGF-C - and VEGF-C +)
-Figure 7D – In the western blot data. Indicate VEGF-C beside the time points to show what factor was used during that specific stimulation study
-Figure 9 E – there is expression of Prox1 in the merged image, however in the image E''- there is no PROX1 expression?

Thank you for carefully reading our manuscript and for the suggestions. We have made all the necessary edits.

Reviewer 2

The lymphatic system is a key component of the circulation system and plays various important functions in animals. Malformation of lymphatic vasculature often leads to lymphedema. Unlike development of many other organs/systems, the mechanisms that regulate lymphatic vascular development remain largely unclear. In the manuscript by Cha et al., the authors report a novel regulatory loop that controls the development of lymphatic valves, lymphovenous valves, and venous valves during mouse embryonic development.

Before this work, it has been reported that PROX1, the master regulator of lymphatic system, and the VEGF-C/VEGFR3 signaling pathway form a regulatory loop. This regulatory loop acts in the lymphatic endothelial cells to regulate lymaphtic vascular development. At the molecular level, how this regulatory loop operates remain unknown. Results presented here argue that YAP and TAZ

© 2020. Published by The Company of Biologists under the terms of the Creative Commons Attribution License (https://creativecommons.org/licenses/by/4.0/).

are critical mediators that act downstream of VEGF-C signaling to regulate the expression of PROX1 expression during lymphatic vascular development.

The conclusion of the paper is convincing, supported by high quality results. The manuscript is carefully drafted. In my opinion, this work represents an important contribution the field.

Reviewer 2 Comments for the Author:

Before this work, it has been reported that PROX1, the master regulator of lymphatic system, and the VEGF-C/VEGFR3 signaling pathway form a regulatory loop. This regulatory loop acts in the lymphatic endothelial cells to regulate lymaphtic vascular development. At the molecular level, how this regulatory loop operates remain unknown. Results presented here argue that YAP and TAZ are critical mediators of the PROX1 expression in response to VEGF-C signaling during lymphatic vascular development. The authors started from analyzing the expression/activity of YAP and TAZ during valve maturation. Results show that the activity of YAP and TAZ gradually increases in developing LVs and LVVs. Motivated by this finding, the authors used genetic approach to conditionally and constitutively delete YAP/TAZ in the LEC progenitors. By analyzing the morphology of the mutant embryos, performing IF for markers, and SEM, they found YAP and TAZ are not required for the initiation of lymphovenous valves, but play an important role in their maintenance. To figure out the molecular mechanism by which YAP and TAZ regulate valve development, they carried out analyses in Human Lymphatic Endothelial Cells (HLECs). RNAseq analysis shows that several pathways critical for vascular development were affected by pharmaceutical inhibition of YAP/TAZ. Regulation of Prox1 YAP/TAZ was further validated by siRNA-based knockdown experiment.
Furthermore, the authors identified a TEAD4 binding sequence from the regulatory element of the prox1 gene and validated it by ChIP assay. To determine if YAP/TAZ regulate Prox1 in vivo during mouse lymphatic vascular development, they assessed Prox1 expression in the LECs and LVV-ECs. Indeed, downregulation of Prox1 was detected in mutant embryos deficient in YAP/TAZ. Moreover, deletion of YAP and TAZ aggravates the lymphatic vascular defects associated with Prox1-heterozgosity. The authors further studied the link between YAP/TAZ and VEGF-C signaling during lymphatic vascular development. Results show that in both HLECs and HEK293T cells, VEGF-C induces phosphorylation and nuclear localization of YAP, and the expression of PROX1 at both RNA and protein levels. By analyzing VEGF-C mutants, the expression of YAP/TAZ targets is downregulated in the LVs of Vegfc+/- embryos and VEGF-C regulates the formation of LVVs. Based on these findings, the authors conclude that YAP and TAZ maintain PROX1 expression in the lymphatic vasculature in response to VEGF-C signaling.

Overall, the key finding of this work is novel. Conclusion of the paper is supported by solid experimental evidence. The manuscript is organized and clearly written. I very much like this work. I have only one minor point. I would suggest the authors to quantify the western blot in Figure 7A and present a bar graph.

Thank you for your generous remarks. We have now edited Figure 7A and have provided a quantification for the western blots.

Reviewer 3
The paper by Cha et al investigates the role of YAP and TAZ in lymphatic development using mice. They initially utilise a Lyve1-Cre mediated model of YAP/TAZ knockout that deletes YAP/TAZ from approximately E10.5 onwards and show that YAP/TAZ are essential for vessel morphogenesis during dermal lymphangiogenesis and the formation of LVVs. They show that in the absence of YAP/TAZ, lymphatic vessels have lower levels of PROX1 than normal in developing embryos. In vitro, they go on to show that either using pharmacological or knockdown approaches in LECs, loss of YAP/TAZ function leads to reduced expression of PROX1 and a number of other vascular markers. They also showed that TEAD4 (DNA binding Yap-cofactor) binds upstream of the PROX1 transcription start site and that activation of YAP/TAZ leads to increased PROX1 and YAP/TAZ Target genes. Genetic interaction data is also presented and suggested to indicate genetic interactions between YAP/TAZ, PROX1 and VEGFC/VEGFR3 at the level of different lymphatic phenotypes. In vitro epistasis data suggests that VEGFC increases PROX1 levels in a YAP/TAZ dependent manner and that VEGFC driven proliferation is YAP/TAZ dependent. Importantly, they show that VEGFC+/-

© 2020. Published by The Company of Biologists under the terms of the Creative Commons Attribution License (https://creativecommons.org/licenses/by/4.0/).

heterozygous animals show reduced Prox1 levels and reduced YAP/TAZ target genes. This suggests that YAP/TAZ are essential downstream of VEGFC including in the activation of PROX1.

Overall, the work utilises an impressive array of genetic models and tools. It is quite important as it is proposing a very different model to that recently published by the Koh laboratory (Cho et al 2019). In fact, the previous work suggests that YAP/TAZ repress PROX1, while this study suggests they promote PROX1 expression – quite different findings indeed. On this point, it is important that the authors take the time to highlight the technical differences between the two studies and provide a reasonable explanation for the differences (see below). Overall, the data here are generally quite convincing and so this may significantly clarify our understanding of how Hippo signalling controls lymphatic development.

Reviewer 3 Comments for the Author:

The paper covers a lot of territory and with the many experimental approaches used, there are several technical concerns throughout that the authors should address. This reviewer is also concerned about the accuracy of a few of the major conclusions that are being drawn and whether they are fully supported by the data (see below). In general, the work could benefit from more explanation of experimental detail throughout. Nevertheless, it is overall an interesting and important study that will change our view of how the Hippo pathway and YAP/TAZ function in lymphatic development if a number of issues are addressed.

Concerns to be addressed:

1. The data in Figure 3 is strikingly different to the data shown in the previous paper by Cho et al 2019. In that study, the authors knocked out YAP and TAZ with Prox1-CreErt2 and found a strong up- regulation of PROX1 in large dilated and dysmorphic dermal lymphatics by E15.5. Here the authors see relatively minor defects by E16.5 and potentially a decrease in PROX1 in dermal lymphatics. As the only difference seems to be the driver Cre strain, could the difference be due to inefficient deletion in the Lyve1-Cre strain? Have the authors validated directly that their Lyve1-Cre deletor is efficiently knocking out both YAP and TAZ? If so, they need to show the data. In this context, it is interesting to note that when the authors did use a Prox1-Cre strain in Figure 6 and Supp 3 combined with their KO line, they did start to see the same sort of structural defects reported by Cho et al 2019. Finally, as well as the technical issue above, the paper would be further improved if the authors provided more discussion on the experimental differences that could explain the different conclusions in the two papers.

Thank you for your suggestions.

We have indeed confirmed that *Lyve1-Cre* efficiently deletes YAP/TAZ in LECs. We have added this data as **Supplementary Figure 2**. In addition, we have shown in **Figure 6F** that the expressions of YAP/TAZ and their target genes are downregulated in LECs sorted from *Yap/Taz*^{LECKO} embryos. However, it is possible that Prox1-CreERT2 used by Cho et al is able to more rapidly delete *Yap/Taz*. We have now added the following paragraph to the *Discussion* to address the differences between our findings and that of Cho et al.

>>>> In contrast to our findings Cho et al showed that YAP and TAZ inhibit PROX1 expression in the developing lymphatic vessels (Cho et al., 2019). The differences could be due to the distinct Cre lines that were used. While we used *Lyve1-Cre*, Cho et al used Prox1-CreERT2 (Bazigou et al., 2011). While Cho et al observed severe edema in their mutant embryos we did not observe edema in most of our samples. The more severe phenotype observed by Cho et al could be due to more potent/rapid gene deletion by Prox1-CreERT2. As mentioned above we suspect that YAP/TAZ play a primary role in maintaining the integrity of LVs and LVVs. Degeneration of LVVs at an earlier stage in the *Yap/Taz*^{LECKO} embryos generated by Cho et al might have affected lymphatic drainage earlier, which in turn would have prevented lymphatic vessel maturation resulting in sustained expression of PROX1. Deleting *Yap/Taz* at various developmental time points and in specific compartments of the lymphatic vasculature (LEC progenitors, LECs, LVs, LVVs, tip cells, stalk cells) could test these possibilities and provide better resolution of YAP/TAZ activity.

© 2020. Published by The Company of Biologists under the terms of the Creative Commons Attribution License (https://creativecommons.org/licenses/by/4.0/).

There are also differences in in vitro data generated by Cho et al and us. While, we have determined that inhibition of YAP/TAZ activity by VP or siRNA inhibits PROX1 expression, results generated by Cho et al make the opposite conclusion. It is possible that these differences are due to differences in cell lines or culture conditions that were used. Nevertheless, there are important points of congruity between our findings and Cho et al. Specifically, we also found that over-activation of YAP/TAZ, via pharmacological inhibition of MST1/2 or RNAi-mediated depletion of LATS1/2 reduces PROX1 expression in confluent HLECs. These results—both loss and over-activation of YAP/TAZ could result in the down regulation of PROX1 expression in the lymphatic vasculature—suggest that a precise level of YAP/TAZ activity regulates PROX1 expression.

2. The increase of Endomucin in the lymph sacs of Prox1-Cre YAP/TAZ KO mice may be indicative of a change in LEC fate to a blood vascular like fate. However, this is a big claim to be based on just one marker. Can the authors confirm this with additional markers? Is this phenotype present earlier than E14.5 and if so doesn't this suggest that in fact YAP/TAZ do play a role in specification and LEC fate but that the late deletion using Lyve1-Cre is the reason for no obvious early specification defects? A more clear discussion of the timing of knockout relative to key events in mouse lymphatic development is needed.

As the reviewer may be aware *Prox1*$^{+/Cre}$ mice have reduced survival, which is even more aggravated in *Prox1*$^{+/Cre}$;*Yap*$^{+/f}$;*Taz*$^{+/f}$ background. Therefore, we analyzed only limited number of markers at one developmental time point (E14.5). We chose PROX1, VEGFR3 and endomucin as these are the best markers to distinguish blood endothelial cells from LECs. LEC marker VEGFR3 appears to be normally expressed in these mutants although PROX1 expression is downregulated. Endomucin is expressed in a subset of LECs in mutants. As stated in the previous version of the manuscript "This observation suggests that the LECs of *Prox1*$^{+/Cre}$;*Yap/Taz*^{LECKO} embryos have abnormally acquireda partial blood vascular endothelial cell identity". However, we agree with the reviewer's concern and we have now toned-down our conclusion as

>>>This observation suggests that a subset of LECs in*Prox1*$^{+/Cre}$;*Yap/Taz*^{LECKO} embryos have abnormally acquired a partial blood vascular endothelial cell identity.

We have also revised the final sentence of this section as

>>>Overall, our data reveal that genetic reduction of *Prox1* together with *Yap* and *Taz* results in the partial loss of LEC identity and improper morphogenesis of lymphatic vessels.

3. The authors conclude that "our data suggest that YAP and TAZ are not required for the initial specification of LECs" however they delete from E10.5 well after the initial specification of LECs at the level of the CV. They would need to delete before specification to test the hypothesis that YAP/TAZ influence specification. As such they should revise their conclusion about specification or perform additional analyses (as point 2).

We have now determined that YAP/TAZ are indeed significantly downregulated in the LECs at E14.5 (**Supplementary Figure 2**). In addition, we do not observe CTGF expression in non-valvular LECs. Hence, it seems unlikely that YAP/TAZ are required for the specification of LECs. Nevertheless, as we are now focusing on the roles of YAP/TAZ in valve development (please see our response to your comments below), and as we have not demonstrated that YAP/TAZ expression is lost from all LECs during specification, we have removed our statements about specification as suggested.

4. In Figure 3 H vs I Prox1 expression looks to be at normal levels in panel I except for the failure of the lymphatics to form valve territories (which express high PROX1). If fluorescence intensity is quantified in the KO LEC nuclei vs non valve territory LECs, is there a change in expression levels that fits with the in vitro suggestion of YAP-TAZ regulating PROX1? (obviously any quantification would need to be normalised and control for variation in staining from embryo to embryo)

Thank you for this insightful comment.

As the reviewer correctly points out it is immensely clear that PROX1high territories (valves) are lost in the mutant embryos. PROX1 expression in non-valvular LECs near LVVs is indeed reduced

© 2020. Published by The Company of Biologists under the terms of the Creative Commons Attribution License (https://creativecommons.org/licenses/by/4.0/).

(Figure 6C). However, PROX1 expression in the dermal lymphatic vessels is not downregulated **(Figure 6G)**. Hence, the primary role of YAP/TAZ is likely to maintain PROX1 expression in the valves. This conclusion is supported by the fact that YAP/TAZ and CTGF expressions are enriched in the valves.

5. The claim that "the transcriptional co-activators YAP and TAZ are required to maintain PROX1 expression in response to VEGF-C signalling" seems to be an overstatement. This also impacts the title of the paper. The authors conclude that "Together these results suggest that YAP/TAZ are necessary to maintain PROX1 expression in developing lymphatic vasculature" in relation to the analysis of Prox1-tdTomato levels in Figure 5. The reduction in Prox1 levels is really quite subtle – the graph in figure 5 shows a reduction by about ~30% based on q-PCR and this is less of a reduction than for YAP/TAZ targets. Based on that, a fair conclusion looking at this data would be that Prox1 is still maintained in the absence of YAP/TAZ at both the protein and the transcriptional level but that VEGFC enhances PROX1 expression in a YAP/TAZ dependent manner.

Thank you again for this comment.

As discussed above the role of YAP/TAZ in maintaining PROX1 expression in the valves is most obvious. It is also clear that YAP/TAZ activity is enriched in the valves. Correspondingly, we have revised the title of the manuscript to reflect these conclusions.

>>YAP and TAZ maintain PROX1 expression in the developing lymphatic and lymphovenous valves in response to VEGF-C signaling

I hope this is acceptable to the reviewer.

6. How specific is the response to the drug VP in cultured LECs? Are non-Hippo targets unchanged? How many genes overall were changed in the RNA seq analysis and were pathways associated with cell death or drug toxicity changed? A full bioinformatic analysis and QC should be provided in supp material with the RNA seq data to give the reader confidence that the analysis was clean and the observations presented are specific.

We are now providing the entire RNA-seq data set as **Supplementary Table 1**. Based on the Log_2(fold change) >1 that we set for differentially expressed genes we determined that 794 genes were upregulated and 2161 genes were downregulated by VP. The canonical Hippo target gene CTGF is significantly downregulated by VP. However, due to the large number of genes that are differentially expressed we are not making the conclusion that VP is specifically targeting Hippo signaling. We have nevertheless verified that siYAP/TAZ is downregulating CTGF and the primary gene of our focus PROX1 (**Figure 5**). We hope that the reviewer finds these results satisfactory.

© 2020. Published by The Company of Biologists under the terms of the Creative Commons Attribution License (https://creativecommons.org/licenses/by/4.0/).

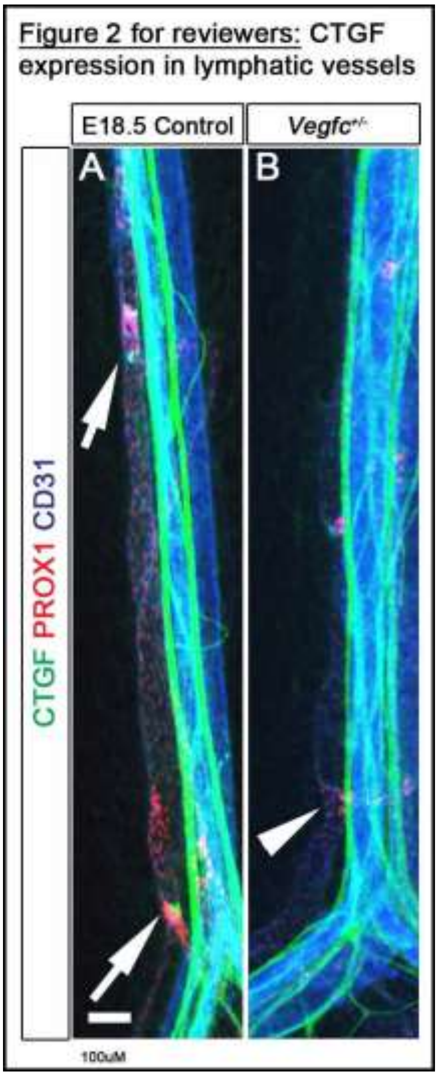

7. For immuno-fluorescence stains such as in Supp Figure 1; it is very difficult to appreciate if the staining for YAP/TAZ or CTGF is specific. Can the authors show a wider field of view for the stains to indicate if there is tissue specificity? How were these controlled for specificity?

We are providing the lower magnification picture of CTGF IHC here for the reviewer (**Figure 2 for reviewers**). As you can see it localizes quite specifically to the LVs. This data is consistent with the results of Sabine et al., JCI 2015. Please also note the weaker PROX1 and CTGF expressions in *Vegfc*$^{+/-}$ sample. If the reviewer finds it necessary we will add it as a supplementary figure to the manuscript.

We have also added a new figure (**Supplementary Figure 2**) in which we have performed YAP/TAZ IHC in *Lyve1-Cre;Yap*$^{f/f}$*;Taz*$^{f/f}$ embryos. This data should hopefully clear the concerns about this antibody.

8. Figure 8 seems to lack careful quantification of the phenotypes, making it hard to interpret how representative the images provided are. N- values for the number of embryos examined are given, but how many LVV forming cells were scored per embryo of each genotype is unclear. Likewise, for dermal lymphatics and venous valve cells. Further quantification is needed to strengthen the data.

We apologize for the confusion. 2 LVV/venous valve-complexes are present in every embryo. The panels show one such complex. We analyze at least 3 embryos per genotype and we analyze both LVV/venous valve-complexes in every embryo. E, F show the entire back skin of embryos. Therefore, these panels are representative of 3 skins from 3 embryos.

© 2020. Published by The Company of Biologists under the terms of the Creative Commons Attribution License (https://creativecommons.org/licenses/by/4.0/).

We have revised the legend as following.

>>> Statistics: (A-D, G, H) n=3 embryos per genotype and 2 LVV/venous valve-complex per embryo; (E, F) n=5 dorsal skins from 5 embryos per genotype.

9. In Figure 1, the Taz staining looks pan-endothelial. Is this the case? Or is there quantifiably some restriction or enrichment in the valve domains? Please show evidence of restriction to valves if that is the claim.

TAZ is expressed in all endothelial cells. Nevertheless, within the lymphatic vessels TAZ is enriched in valvular endothelial cells. Furthermore, the YAP/TAZ target CTGF is specifically expressed in valves as reported previously by Sabine et al., *JCI* 2015.

10. Is Lyve1 really exclusively a LEC marker in ECs? If not, then the double KO model should not be referred to as LECKO and expression in other vessels, such as veins, should be noted clearly as a caveat to these analyses.

As we and others have reported in the past that *Lyve1-Cre* is expressed in the cardinal vein from which most LECs originate. This is reason for the loss of venous valves in *Lyve1-Cre;Yap$^{f/f}$;Taz$^{f/f}$* embryos. Despite this fact Lyve1-Cre activity is reasonably specific to the LECs in the dermal vasculature. However, as indicated by the reviewer *Lyve1-Cre* is indeed expressed in the blood endothelial cells of tissues such as the gut and lungs. This is why we restricted our original analysis of *Lyve1-Cre;Yap$^{f/f}$;Taz$^{f/f}$* embryos to the dermis. We have now added the data from the gut of the mutant embryos as a supplementary figure and highlighted the limitation of the *Lyve1-Cre* as suggested.

11. There is no legend for Figure 2I and so it is unclear what was scored here (number of ECs per valve? Number of valves per embryo?) and what the graph represents.

We apologize for our oversight. We have now added the legends.

>>>Every embryo has 4 LVVs. We analyzed n=4 embryos/genotype/stage and calculated the average number of LVVs per embryo.

12. Statistics – the replicates are given at the end of each figure legend but it is unclear if the replicates are technical or biological, particularly for in vitro data. Please clarify in detail in the methods.

All replicates are independent experiments. We have stated it explicitly in the revised *Material and Methods*.

>>>>For biochemical analysis n indicates the number of times the experiments were independently performed and for histological analysis n indicate the number of embryos analyzed per genotype. All experiments were performed at least three times or more.

Second decision letter

MS ID#: DEVELOP/2020/195453

MS TITLE: YAP and TAZ maintain PROX1 expression in the developing lymphatic and lymphovenous valves in response to VEGF-C signaling

AUTHORS: Boksik Cha, YenChun Ho, Xin Geng, Md Riaj Mahamud, Lijuan Chen, Yeunhee Kim, Dongwon Choi, Tae Hoon Kim, Gwendalyn J Randolph, Xinwei Cao, Hong Chen, and R. Sathish Srinivasan

© 2020. Published by The Company of Biologists under the terms of the Creative Commons Attribution License (https://creativecommons.org/licenses/by/4.0/).

ARTICLE TYPE: Research Article

I am happy to tell you that your manuscript has been accepted for publication in Development, pending our standard ethics checks.

Reviewer 1

Advance summary and potential significance to field

The data presented herein expand our understanding of the role YAP/TAZ play in maintaining Prox1 expression and its resulting affects on the Prox1-VEGF-C feedback loop involved in LV and LVV development. Moreover, the authors show that VEGF-C is required for LV development. Considering the depth and quality of the experiments, these studies provide important contributions on YAP/TAZ functions in LV and LVV development.

Comments for the author

The authors appropriately and reasonably addressed my previous concerns. I have no additional remarks or concerns.

Reviewer 2

Advance summary and potential significance to field

This is a very nice work. The authors have addressed my previous concern. I would like to recommend it for publication.

Comments for the author

The authors have addressed my previous concern. I would like to recommend it for publication.

Reviewer 3

Advance summary and potential significance to field

The manuscript by Cha et al presents extensive new data on the role of YAP and TAZ in lymphatic development. They convincingly show that YAP/TAZ promote Prox1 expression in valve territories and positively impacts Prox1 expression downstream of Vegfc. The revision of this manuscript provides further analysis and data at several places that clearly improve the work overall. There are also reasonable rebuttals of some reviewer comments. It is a good revision overall and this reviewer has no further concerns.

Comments for the author

No further issues.

© 2020. Published by The Company of Biologists under the terms of the Creative Commons Attribution License (https://creativecommons.org/licenses/by/4.0/).
